# Microbial Communities in Agave Fermentations Vary by Local Biogeographic Regions

**DOI:** 10.1111/1758-2229.70057

**Published:** 2025-01-24

**Authors:** Angélica Jara‐Servin, Luis D. Alcaraz, Sabino I. Juarez‐Serrano, Aarón Espinosa‐Jaime, Ivan Barajas, Lucia Morales, Alexander DeLuna, Antonio Hernández‐López, Eugenio Mancera

**Affiliations:** ^1^ Laboratorio de Genómica Ambiental, Departamento de Biología Celular, Facultad de Ciencias Universidad Nacional Autónoma de Mexico Ciudad de México Mexico; ^2^ Departamento de Ingeniería Genética, Centro de Investigación y de Estudios Avanzados del Instituto Politécnico Nacional Unidad Irapuato Irapuato Mexico; ^3^ Escuela Nacional de Estudios Superiores Unidad León Universidad Nacional Autónoma de México León Mexico; ^4^ Laboratorio Internacional de Investigación Sobre el Genoma Humano Universidad Nacional Autónoma de México Juriquilla Mexico; ^5^ Unidad de Genómica Avanzada Centro de Investigación y de Estudios Avanzados del Instituto Politécnico Nacional Irapuato Mexico

**Keywords:** agave fermentation, metabarcoding, microbial communities, open fermentation

## Abstract

The production of traditional agave spirits in Mexico, such as mezcal, involves a process that uses environmental microorganisms to ferment the cooked must from agave plants. By analysing these microorganisms, researchers can understand the dynamics of microbial communities at the interface of natural and human‐associated environments. This study involved 16S and ITS amplicon sequencing of 99 fermentation tanks from 42 distilleries across Mexico. The Agave species used, production methods, climatic conditions and biogeographic characteristics varied significantly among sites. However, certain taxa were found in most fermentations, indicating a core group of microorganisms common to these communities. The primary variable consistently associated with the composition of both bacterial and fungal communities was the distillery, suggesting that local production practices and site‐specific attributes influence the microbiomes. The fermentation stage, climate and producing region also affected community composition but only for prokaryotes. Analysis of multiple tanks within three distilleries showed taxa enriched in specific fermentation stages or agave species. This research provides a detailed analysis of the microbiome of agave fermentations, offering important knowledge for its management and conservation.

## Graphical abstract

We performed a country‐wide metabarcoding analysis of the microbial communities associated with the fermentations used for the production of artisanal agave spirits in Mexico. Despite the biogeographic and cultural differences of the producing sites, we identified a core of bacteria and fungi that are common in most fermentations. Our results also suggest that the microbial composition of these communities is locally determined at the distillery level.

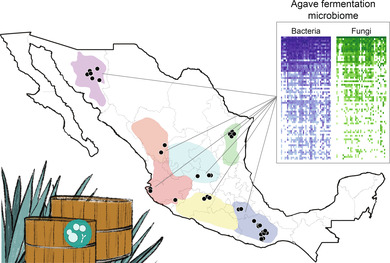

## 1 Introduction

Mexico stands out for its biological and cultural diversity, a result of its intricate geological and social history. For example, it has one of the world's highest numbers of reptile, oak and pine species (Farjon 1996; Nixon 2006; Suazo‐Ortuño, Ramírez‐Bautista, and Alvarado‐Díaz 2023). Additionally, it is one of the most multilingual regions, and its population is predominantly admixed (Eberhard, Simons, and Fennig 2020; Sohail et al. 2023). However, we know relatively less about its microbial diversity in both natural and human‐associated habitats. The production of spirits from agave plants is a highly ingrained activity in Mexico. It is yet unclear whether this practice dates to pre‐Columbian times (Machuca Chávez 2018; Ojeda‐Linares et al. 2021), but currently, it is performed from the southernmost provinces of Mexico to the northern states that border the USA (Serra Puche and Lazcano Arce 2016; Cabrera‐Toledo et al. 2022). Its widespread distribution is probably related to the abundance of agave plants throughout the Mexican territory as this region is the centre of the diversity of the genus (Trejo et al. 2018).

*Tequila* is arguably the best known and most commercial of the agave spirits, nowadays produced in highly industrial settings. However, most other agave distillates, such as *mezcal*, *raicilla* or *bacanora*, are produced in a more artisanal way that is characterised by open, ‘spontaneous’ fermentations in which the producers do not rely on an established inoculum of microorganisms (Arellano‐Plaza et al. 2022). Instead, the cooked and macerated agave hearts are fermented by microorganisms coming from the surrounding environment. Therefore, the characterisation of the microbiome involved in agave fermentations is not only essential to better understand the production of these beverages of traditional and commercial importance, but it also provides insight into the natural communities of microorganisms in Mexico.

The vast area where agave spirits are produced—a region that is larger in size than Western Europe—shows a wide range of environmental conditions and production practices (Arellano‐Plaza et al. 2022). For instance, there are distilleries located almost at sea level, while others stand at 2000 m above it, encompassing arid, semi‐arid and subhumid climates in subtropical and tropical areas. This results in a wide range of temperatures and annual rainfalls determining, among other things, the agave species that can grow in each region. Over 50 different species of agave have been reported to be used for the production of agave spirits and their sugar, nitrogen and other metabolite content could vary among them (Colunga‐GarciaMarin et al. 2017). The cooking process hydrolyses fructans, the most abundant carbohydrates in agave plants, releasing free sugars such as fructose, for the fermentation step (values from 20 g/L to 295 g/L of sugar have been reported (Colón‐González et al. 2024)). However, cooking also produces compounds that can inhibit microorganism growth such as 5‐hydroxymethylfurfural and furfural (Mancilla‐Margalli and López 2002). Together with metabolites from the agave plants such as saponins, these compounds can make agave fermentation a challenging environment for microorganisms. For an in‐depth review of agave fermentations as an ecological environment for microorganisms, see (Colón‐González et al. 2024).

The most common microorganisms identified in agave fermentations using culture‐based microbiological methods, with their intrinsic biases, are ascomycetous yeasts and lactic acid bacteria (Escalante‐Minakata et al. 2008; Kirchmayr et al. 2017; Gallegos‐Casillas et al. 2023; Colón‐González et al. 2024). Previous studies have mainly focused on yeasts since they are thought to be the main contributors to ethanol production. The most extensive characterisation to date of the fungal communities of traditional agave fermentations recently revealed a core of six ascomycetous yeasts that were commonly isolated from this fermentation (Gallegos‐Casillas et al. 2023). The six species had also been isolated in previous studies, even when they were only carried out in a handful of factories mostly located in the state of Oaxaca, the main mezcal‐producing state (Kirchmayr et al. 2017; Nolasco‐Cancino et al. 2018). The different species of yeast involved in these fermentations are not only thought to contribute to ethanol production but also to the synthesis of other compounds with organoleptic properties.

Much less is known about the prokaryotic microbiome of agave fermentations and bacteria in other anthropogenic fermentations are often considered contaminants that can lead to spoilage. The four previous studies that analysed prokaryotes in traditional fermentations for the production of agave spirits identified not only species of lactic acid bacteria but also acetic acid bacteria and spore‐forming bacteria (Escalante‐Minakata et al. 2008; Narvaez‐Zapata et al. 2010; Kirchmayr et al. 2017, 2024). The ethanol‐producing capabilities of some of these species suggested that bacteria could actually have a more important role than previously thought in the production of traditional agave spirits (Kirchmayr et al. 2017). Despite the previous efforts mainly using culture‐based microbiological methods, there are still many basic open questions regarding the microbiome of traditional agave fermentations. In fact, it is unclear whether the isolated yeast species are indeed the predominant fungi in these fermentations. It is possible that other fungi are also abundant but are not commonly isolated with classical microbiological methods. The types of bacteria that play a role in the different regions where agave spirits are produced are also unknown.

To get deeper insights into both the bacterial and fungal microbiome of agave fermentations, we performed 16S rRNA gene (16S) and ribosomal internal transcribed spacer (ITS) amplicon sequencing from must samples collected from 99 fermentation tanks from 42 different distilleries throughout the regions where agave spirits are produced in Mexico. Our results showed that despite the diversity in production practices and biogeographical parameters, there is a core of species that defines the microbial communities throughout the country. Many of the taxa coincide with species previously isolated from these fermentations, but we also identified prevalent species that had never been associated with agave fermentations before. Although Ascomycota were by far the most common fungi, we also identified fungi from five other phyla, showing overall considerable fungal diversity. The bacterial microbiome is even more diverse than the fungal component, and it is dominated by species of lactic acid bacteria. The only association of the composition of the microbiome that we observed in both bacteria and fungi was with the distillery to which the samples belong. To our knowledge, our work represents the first country‐wide analysis employing metabarcoding approaches of the microbiome of the fermentations used for traditional agave spirit production, considerably expanding our understanding of these communities.

## 2 Materials and Methods

### 2.1 Collection of Agave Fermentation Samples, DNA Extraction and Sequencing

**FIGURE 1:**
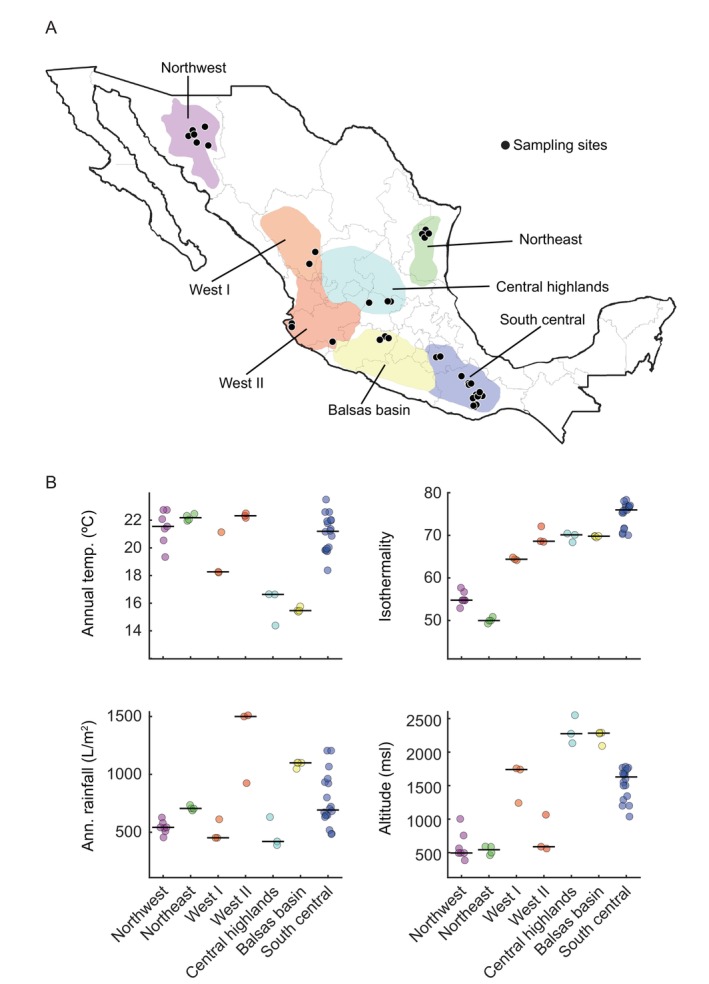
Distribution and characteristics of the agave spirit distilleries. To be able to compare the data with our previous work, the format of the map and the boxplots of the climatic characteristics are the same as in Gallegos‐Casillas et al. (2023). However, the data in the figures correspond to the properties of the distilleries employed in the current work. (A) Map of Mexico showing the geographical location of the 42 distilleries (black dots) from which the 99 fermentation tanks were sampled in the corresponding producing region which are indicated as polygons in different background colours. Producing regions were defined according to Aguirre, Illsley, and Larson (2006) mostly based on the agave species and production practices employed. The West I and West II regions were considered a single region (West) in the original publication, but after consulting with the authors, the region was divided to take into account the differences that we observed in the production practices of the distilleries of those areas (Gallegos‐Casillas et al. 2023). (B) Climatic characteristics of the locations of the agave fermentation tanks. (top left) Average annual temperature, (top right) isothermality, (bottom right) average annual rainfall and (bottom left) height above mean sea level.

Agave fermentation samples were taken as part of the field work performed by the YeastGenomesMx consortium throughout the seven regions where agave spirits are produced in Mexico (Figure 1). As described before (Gallegos‐Casillas et al. 2023), for each sample, 4 mL of agave ferment was taken with a sterile serological pipet at arm's reach from each of the 99 sampled tanks and was stored in cryogenic vials to be immediately frozen in liquid nitrogen. In several distilleries, more than one fermentation tank was sampled. Associated metadata (Table S1) was collected in the field for each sample, as previously described (Gallegos‐Casillas et al. 2023). At the laboratory, samples were stored at −80°C until processing. Total DNA was extracted using the ZymoBIOMICS DNA Miniprep Kit (D4300) from Zymo Research. Extractions were performed from 500 μL of sample following the manufacturer protocol but adding a 15‐min incubation step at 70°C after lysis and before centrifugation and filtration to improve DNA extraction. DNA quantity, purity and integrity were monitored with a Nanodrop, fluorometry in a Qubit, and by performing agarose gel electrophoresis. 16S rRNA gene and ITS library preparation and sequencing were performed by Zymo Research in an Illumina MiSeq platform and generating a minimum of 25,000 PE 300 bp reads per amplicon type for each sample. PCR amplification was part of the library preparation procedure and was performed by Zymo Research with custom parameters and primers. pPNA and mPNA blockers were used to avoid plant DNA amplification and sequencing.

### 2.2 16S rRNA Gene and ITS Region Amplicons Sequence Processing

Access to the detailed protocol and bioinformatic methods used to process and analyse 16S rRNA genes and ITS amplicon sequences can be found on GitHub (https://github.com/ajaraservin/mezcal). Amplicon data from the pulque microbiome were obtained from publicly available raw reads (Rocha‐Arriaga et al. 2020) so that they could be processed using the same computational pipeline. Quality assessment was performed on all 16S libraries using FastQC v0.12.0 (https://www.bioinformat ics.babraham.ac.uk/projects/fastqc/) and eight samples were discarded due to the low number of reads obtained. This led to one of the distilleries being also excluded from the analysis as there were no samples left from it. The CASPER v0.8.2 assembler was used to merge the pair‐end reads of the V4 region (Kwon, Lee, and Yoon 2014). QIIME's identify_chimeric_seqs.py using ChimeraSlayer (Caporaso et al. 2010) script with BLAST fragments was used to identify chimeric sequences. All samples were concatenated and clustered into operational taxonomic units (OTUs) using a 97% identity threshold with cd‐hit‐est (Li and Godzik 2006). Taxonomy assignment was performed using QIIME's scripts (Caporaso et al. 2010) against the Silva v 138 database (Yilmaz et al. 2014). After singleton, chimera and contamination screening, the remaining sequences were aligned, and a phylogenetic tree was constructed using FastTreeMP v2.1.11 (Price, Dehal, and Arkin 2009).

ITS pair‐end reads were merged using the CASPER assembler and then subjected to a quality check using fastq_quality_filter (Q < 20) from the FASTX‐Toolkit (http://hannonlab.cshl.edu/fastx_toolkit/). Tweleve ITS libraries were discarded due to a low number of reads, which resulted in two distilleries not being included in the analysis. After assembly with PANDASEQ v2.11 and clustering at 97% identity with cd‐hit‐est, the all‐eukaryotes UNITE v8.3 database (Nilsson et al. 2019) was used for taxonomic assignment up to the species level. All chimeras and non‐fungi sequences were discarded using parallel_identify_chimeric_seqs.py and filter_otus_from_otu_table.py scripts (Caporaso et al. 2010).

### 2.3 Diversity and Statistical Analysis

OTUs were used to analyse both the α and β‐diversity of our agave spirit and pulque samples. Phyloseq (McMurdie and Holmes 2013), ggplot2 (Wickham 2016), vegan (Oksanen et al. 2022) and R v4.2.2 default packages (Team 2021) were used for the analysis. Observed, Chao1, Shannon and Simpson diversity indices were calculated to assess α‐diversity, while taxonomic abundance profiles were obtained through clustering at different taxonomic levels. Dendrograms were constructed using the hclust method. Constrained analysis of principal coordinates (CAPs) on an unweighted UniFrac matrix (Lozupone and Knight 2005) of 16S gene sequences was used to evaluate ß‐diversity according to the different variables. Similarly, a Jaccard similarity matrix (Knight et al. 2018) of ITS sequences was used for the PcoA ordination method. UniFrac was not used for ITS analysis given the difficulty of aligning ITS sequences (Halwachs et al. 2017). Both matrices were evaluated using the ANalysis Of SIMilarity (ANOSIM) statistical function (Varsos et al. 2016). The following variables were tested: Distillery (Distillery_ID), Climate (ClimateGroup_GK), Tank temperature (Tank_temperatureC), Fermentation stage (Fermentation_range), Agave species (Agave_speciesname), Region (Region) and Tank material (Tank_material). Variable names in parenthesis are the names employed in Table S1, where a detailed description of each variable is provided. Differential OTUs abundance was estimated using R's package DESeq2 (Love, Huber, and Anders 2014) as has been routinely done (Halwachs et al. 2017). Finally, Geographic Distance Matrix Generator v.1.2.3 (Ersts n.d.) was used to generate a geographic distance matrix for all agave spirit samples. This geographic distance matrix was used to perform a Mantel test (Xia and Sun 2017), using an unweighted UniFrac distance matrix for bacteria and a Jaccard similarity matrix for fungi. Detailed bioinformatic and statistical methods are available at GitHub (/github.com/ajaraservin/mezcal).

## 3 Results

### 3.1 A Country‐Wide Collection of Agave Musts From Diverse Biogeographical Regions

The current work is part of the research performed by the YeastGenomesMx consortium, a group of laboratories interested in the genomic diversity of yeasts and other microorganisms in Mexico. At the time of writing, the consortium had sampled over 90 distilleries in all the regions where agave spirits are produced in Mexico. For the characterisation of the microbiome of agave fermentations described here, we focused on 99 fermentation tanks from 42 distilleries, 67 of which used *Agave angustifolia* , the most used species for traditional agave spirits (Table S1). *A. tequilana* , the agave species used to produce tequila, is by far the most cultivated agave species; however, tequila production is mostly industrial, involving inoculation of the fermentations with commercial yeast strains, and was therefore not considered in this work. Focusing the analysis on distilleries that use *A. angustifolia* allowed us to test the impact of other variables besides agave species on the microbiome of the fermentations, mainly whether there is an association between the location of the distillery and the microbiome. Given that there are regions where *A. angustifolia* is not employed, we also included fermentations of other eight agave species as well as mixtures of two or three species for the production of blended spirits. The species used in these other distilleries are *Agave americana* , *A. durangensis* , *A. inaequidens*, *A. karawinskii*, *A. mapisaga*, *A. potatorum*, *A. rhodacantha* and *A. salmiana* (Table S1). Using this subset that includes fermentations of a variety of agaves, we were also able to test the possible effect of the plant substrate on microbial diversity.

The 42 distilleries are located in 31 different municipalities of the states of Durango, Guanajuato, Michoacan, Oaxaca, Puebla, Tamaulipas, Jalisco and Sonora. In the six first states, the spirit produced is known as *mezcal* while in Jalisco it is known as *raicilla* and in Sonora as *bacanora*. All three spirits have their own designation of origin, although the overall production process is similar. In addition, several producers use the general term ‘destilado de agave’ (Spanish for agave spirit) not to adhere to the official Mexican standards (NOMs) of each of the designations of origin. The two more distant distilleries that were sampled are over 2000 km apart in a straight line, from 29°52′ N and 109°33′ W to 16° N and 96°31′ W. The altitude of the distilleries also varied considerably, from below 500 m above sea level to over 2500 m, and covered climates from tropical to semi‐desertic. Figure 1 shows the geographic location of the distilleries analysed in this study and some of their geographical characteristics.

All but three of the distilleries included in this study used buried stone or brick ovens and the cooked agave hearts were ground either manually, using mills powered by animals or employing motorised mills. We did not measure cooking temperature or duration at each site, but average oven temperatures of 120°C and durations from 3 days to over a week have been reported (Durán‐García, González‐Galván, and Matadamas‐Ortiz 2007). The tanks sampled spanned fermentation times from the day the fermentation was formulated to full maturity when the must was being transferred to the alembic for distillation. Since total fermentation length varies throughout distilleries, this meant that we sampled tanks that had less than a day to those that had been fermenting for close to 2 months. The material of the tanks was diverse including wood, plastic, clay, steel, cement and even cattle hide, but wood was by far the most common followed by plastic among the distilleries sampled. The specific characteristics of the distilleries and fermentation tanks of the sequenced samples are detailed in Table S1.

### 3.2 Bacterial Diversity in Agave Fermentations Across Mexico

In this study, we assessed and compared the bacterial community of agave fermentations throughout Mexico by 16S metabarcode sequencing. Of the 99 sequenced samples, only 91 were sequenced successfully, from which we generated a total of 10,438,060 paired‐end 16S reads. The reads were quality‐filtered and assembled to obtain 4,887,502 sequences with a mean of 53,708.81 ± 15,371.02 sequences per sample (Table S2). These sequences were then clustered using a 97% identity cut‐off into 10,093 OTUs, corresponding to 888 genera that matched a total of 29 distinct prokaryotic phyla. On average, each sample contained a richness of 1002.08 ± 316.47 OTUs. The average expected Chao1 richness was 1690.17 ± 500.67 OTUs which showed that our effort covered a major part of the expected bacterial OTUs. The average Shannon–Weiner (H′) and Simpson's (D) diversity indices across samples were 3.26 ± 0.66 and 0.86 ± 0.12, respectively.

Throughout the samples, the composition of the prokaryotic communities at the phylum level was similar and was predominantly composed of Firmicutes (71.82%), Proteobacteria (14%), Actinobacteriota (4.82%), Bacteroidota (4.02%), Verrucomicrobiota (0.76%) and Patescibacteria (0.65%). From the 71 identified bacterial classes, Bacilli dominated (6802 OTUs; 68.33%) followed far away by Alphaproteobacteria (971 OTUs; 9.75%), Gammaproteobacteria (971 OTUs; 9.75%), Actinobacteria (412 OTUs; 4.13%), Bacteroidia (399 OTUs; 4.00%), Clostridia (373 OTUs; 3.74%) and Parcubacteria (46 OTUs; 0.46%). The 10 most abundant genera were *Weissella* (948 OTUs; 7125.88 ± 59,886 reads per sample), *Paucilactobacillus* (258 OTUs; 7083.38 ± 43,347.79 reads per sample), *Lentilactobacillus* (749 OTUs; 2598.77 ± 22,071.50 reads per sample), *Leuconostoc* (678 OTUs; 6051.23 ± 59,767.16 reads per sample), *Oenococcus* (266 OTUs; 4222.21 ± 28,871.62 reads per sample), *Lactiplantibacillus* (509 OTUs; 4372.17 ± 21,691.45 reads per sample), *Liquorilactobacillus* (373 OTUs; 10,639.12 ± 67,213.38 reads per sample), *Lacticaseibacillus* (587 OTUs; 116.16 ± 384.15 reads per sample), *Acetobacter* (236 OTUs; 1351.17 ± 4614.47 reads per sample) and *Secundilactobacillu*s (572 OTUs; 5501.06 ± 35,204.32 reads per sample). All of them, but *Acetobacter*, are lactic acid bacteria. We also observed considerable diversity within the identified genera. For example, 50.22% of genera (446) was represented by more than one OTU and 10.13% (90 genera) by 10 or more (Table S2).

**FIGURE 2:**
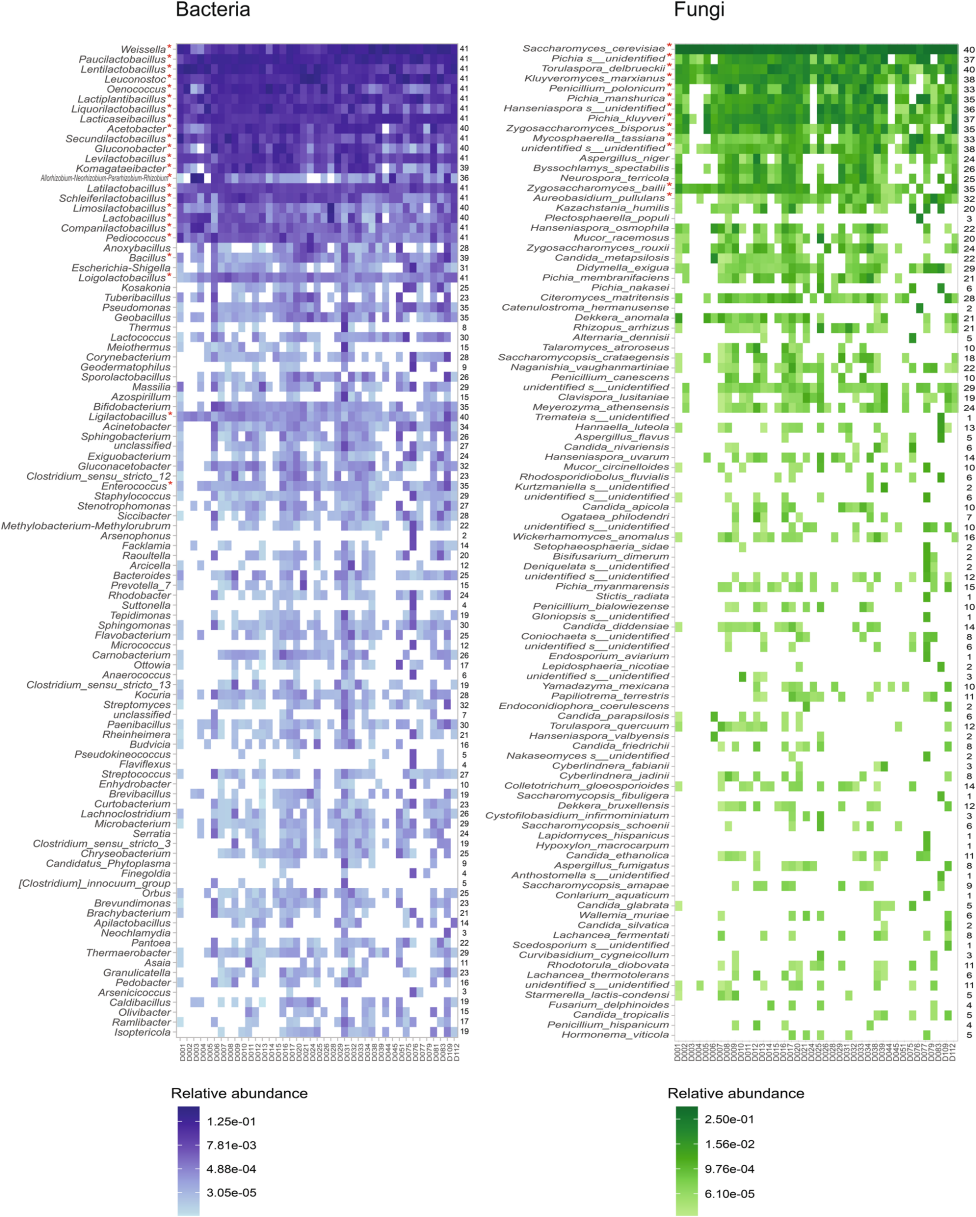
Bacterial and fungal composition of the microbial communities of agave fermentations. Heatmaps showing the relative abundance of the bacterial genera (left panel) and fungal species (right panel) identified in each of the distilleries. Only the 100 most abundant taxa are shown ordered from top to bottom according to their overall relative abundance. Relative abundance refers to the number of reads of a genus/species relative to the total number of reads in the distillery or in all distilleries. Taxa considered part of the core microbiome (present in 80% or more of the distilleries and in all the seven producing regions, Table 1) are marked with a red asterisk. Numbers at the right of each heatmap show the total number of distilleries in which the taxon was identified. All the bacterial and fungal taxa identified are reported in Table S2.

The most abundant genera described above, except *Acetobacter*, were present in all the distilleries that we sampled. Overall, there were 15 genera present throughout the sites analysed, all lactic acid bacteria, that could be considered the core bacterial microbiome of agave fermentations (Table 1). If a lenient definition of the core is used, in addition to these 15, there were 11 other genera that were present in 80% or more of the distilleries and in all the seven agave‐spirit‐producing regions (Table 1). By far, the majority (73%) of the 26 genera were lactic acid bacteria. The taxonomic abundance profile of prokaryotes is shown in Figure 2 and the complete list of genera is provided in Table S2.

**TABLE 1:** Bacterial and fungal core microbiome of traditional agave fermentations.

Bacterial genus^a^	OTUs^b^	Distilleries^c^	Fungal species^a^	OTUs^b^	Distilleries^c^
*Weissella*	948	100	*Saccharomyces cerevisiae*	109	100
*Paucilactobacillus* ^d^	258	100	*Pichia spp*.	24	93
*Lentilactobacillus* ^d^	749	100	*Torulaspora delbrueckii*	24	100
*Leuconostoc*	678	100	*Kluyveromyces marxianus*	14	95
*Oenococcus*	266	100	*Penicillium polonicum* ^d^	16	83
*Lactiplantibacillus*	509	100	*Pichia manshurica*	26	88
*Liquorilactobacillus* ^d^	373	100	*Hanseniaspora spp*.	15	90
*Lacticaseibacillus*	587	100	*Pichia kluyveri*	346	93
*Acetobacter*	236	98	*Zygosaccharomyces bisporus*	13	88
*Secundilactobacillus*	572	100	*Mycosphaerella tassiana* ^d^	11	83
*Gluconobacter*	210	98	*Unidentified unidentified*	32	95
*Levilactobacillus*	349	100	*Zygosaccharomyces bailii*	6	88
*Komagataeibacter*	163	95	*Aureobasidium pullulans* ^d^	8	80
*ANPR* ^d^	34	88			
*Latilactobacillus* ^d^	129	100			
*Schleiferilactobacillus* ^d^	125	100			
*Limosilactobacillus* ^d^	169	98			
*Lactobacillus*	122	98			
*Companilactobacillus* ^d^	144	100			
*Pediococcus*	194	100			
*Bacillus*	56	95			
*Loigolactobacillus* ^d^	44	100			
*Geobacillus*	21	85			
*Bifidobacterium* ^d^	27	85			
*Ligilactobacillus* ^d^	65	98			
*Enterococcus* ^d^	42	85			

Abbreviation: *ANPR*, *Allorhizobium–Neorhizobium–Pararhizobium–Rhizobium*.

^a^ Taxa were included if they were in 80% or more of the distilleries and in all the seven producing regions. They are shown from top to bottom in descending order of relative abundance as in Figure 2.

^b^ Number of OTUs that were classified into the taxon.

^c^ Percentage of distilleries in which the taxon was identified.

^d^ Genera/species that had not been associated with agave fermentations before.

From the 734 bacterial genera that could be classified, 96% had not been previously associated with traditional agave fermentations using culture‐based microbiological methods and including a recent report that used metabarcoding in a single distillery (Colón‐González et al. 2024; Kirchmayr et al. 2024). From the core bacterial microbiome, 12 (46%) genera had not been reported in previous literature (Table 1). It is important to note that some of these genera were recently defined which could explain why they had not been previously reported in agave fermentations. Furthermore, from the 31 genera that had been reported in these fermentations in the past, our survey only missed *Zymomonas*. However, *Zymomonas* is not present as a genus in the most current 16S reference database in which the sequences that belonged to this genus have been reassigned to five genera of the family Sphingomonadaceae, four of which we did identify in agave fermentations. Overall, our results showed that the sampling effort contributed considerably to the understanding of the prokaryotic composition of traditional agave fermentations, a part of this microbiome that had been mostly neglected.

### 3.3 Fungal Diversity in Agave Fermentations Across Mexico

To characterise the fungal microbiome of agave fermentations, we performed amplicon sequencing of the ITS region from the must samples. A total of 87 samples were successfully sequenced yielding a total of 10,199,202 paired‐end ITS reads. The reads were assembled and quality‐filtered to obtain 2,135,405 assembled sequences with an average of 24,544.8 ± 11,380.3 reads per sample (Table S2). By clustering the sequences at a phylogenetic distance of 3%, we identified a total of 1118 ITS OTUs. An average of 92.85 ± 39.72 OTUs was observed in the 87 samples, while the average expected richness (Chao1) was 153.92 ± 70.44. Therefore, as with prokaryotes, our effort covered most of the expected diversity per sample. The diversity in each sample was considerably lower than for bacteria with an average Shannon–Weiner diversity index (H′) of 1.20 ± 0.74 and an average Simpson diversity index (D) of 0.44 ± 0.27. By far, most of the fungal OTUs belonged to the phylum Ascomycota (1035 OTUs; 92.57%), but we also identified Basidiomycota (40 OTUs; 3.57%), Mucoromycota (7 OTUs 0.62%), Chytridiomycota (2 OTUs; 0.17%), Mortierellomycota (1 OTU; 0.08%) and Rozellomycota (1 OTU; 0.08%). Eleven fungal OTUs (0.98%) remained unidentified even at the level of phyla.

The 1118 identified ITS OTUs were distributed in 325 fungal species. The 10 most abundant fungal species were *Saccharomyces cerevisiae* (109 OTUs; 16,793.54 ± 11,282.19 reads per sample), *Pichia manshurica* (26 OTUs; 1159.20 ± 4523.03 reads per sample), an unidentified species of *Pichia* (24 OTUs; 1155.11 ± 3443.42 reads per sample), *Torulaspora delbrueckii* (24 OTUs; 896.75 ± 1997.14 reads per sample), *Penicillium polonicum* (16 OTUs; 686.39 ± 2436.22 reads per sample), *Kluyveromyces marxianus* (14 OTUs; 624.43 ± 1062.53 reads per sample), *Zygosaccharomyces bisporus* (13 OTUs; 535.22 ± 1069.18 reads per sample), an unidentified species of *Hanseniaspora* (15 OTUs; 514.40 ± 953.92 reads per sample), *Pichia kluyveri* (346 OTUs; 496.59 ± 441.87 reads per sample) and another unidentified species (32 OTUs; 295.34 ± 1648.88 reads per sample). These species accounted for 94.26% of the total number of reads. As for many prokaryote genera, large intra‐specific diversity was observed; 81 (23.5%) species had more than two OTUs and 19 (5.5%) > 10 (Table S2).

Only *S. cerevisiae* and *T. delbrueckii* were present in all the distilleries, but there were other 11 fungal species identified in 80% or more of the sites and in all the seven producing regions. These 13 species could be considered the core fungal microbiome of agave fermentations (Table 1). One of the core species could not be identified even at the level of phylum, but the rest are all ascomycetes. Numerous rare species were also detected, with 125 found in only one fermentation tank, and we also identified 35 and 34 fungal OTUs that could not be assigned to any species or genus, respectively. The taxonomic abundance profile of fungi is shown in Figure 2, and the complete list of species is provided in Table S2.

In total, 234 (91.0%) of the identified fungal species had never been associated with traditional agave fermentations before (Colón‐González et al. 2024). From the core species, *P. polonicum* , *M. tassiana* and *A. pullulans* had not been reported previously. On the other hand, 23 species previously isolated using culture‐based methods from traditional agave fermentations were not identified in our survey, although 11 of these are not included in the ITS database used here. At the level of genus, 90.7% of the 184 genera identified here had not been associated with traditional agave fermentations and we only missed three previously isolated genera (15%), all Basidiomycetes, and one of them is not included in the ITS database that we employed. Together, these results show that our effort enriches the understanding of the fungal communities of traditional agave fermentations, adding a considerable number of species and genera that had not been associated with this environment.

### 3.4 Distillery Origin Is the Sole Driver of Overall Microbial Community Composition in Traditional Agave Fermentations

3.4.1

**FIGURE 3:**
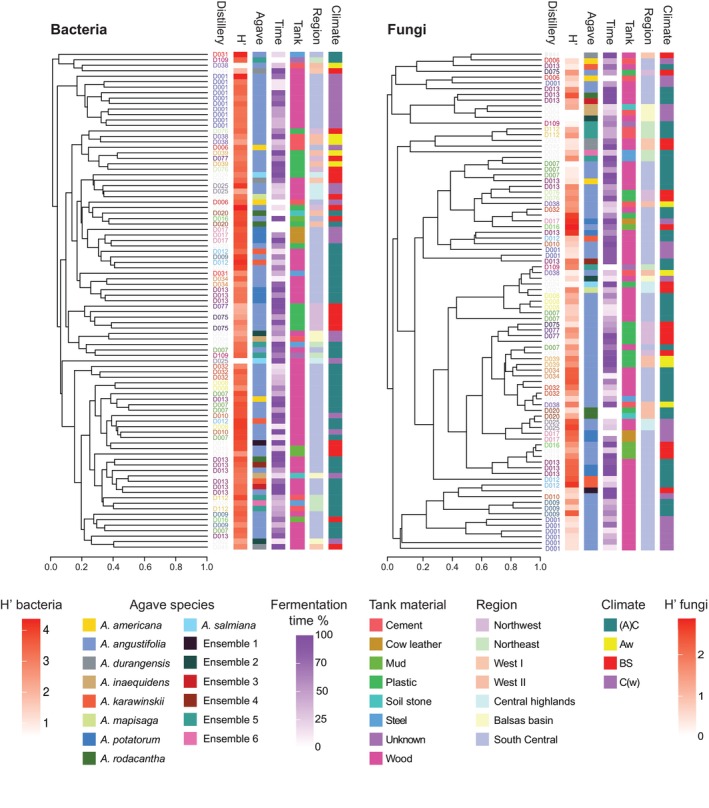
Analysis of the microbial diversity of agave fermentations and its relationship with the production practices and geographical characteristics. Dendrograms show the similarity of the bacterial (left panel) and fungal (right panel) microbiomes of the fermentations sampled. The same font colour is used for samples from the same distillery. UniFrac distance was employed for bacteria while Jaccard similarity was for fungi. The most important production and geo‐climatic characteristics associated with each sample are shown in coloured vertical bars at the right of each dendrogram. From left to right, Shannon diversity index (H′), agave species employed (Agave), fermentation stage expressed as a percentage of the total fermentation time (Time), material of the tank (Tank), producing region as described in Figure 1 (Region) and climate group (Climate) are shown. The composition of fermentations with mixtures of agave plants (ensembles) is described in Table S1. Climate groups according to Köppen and modified by E. Garcia were used (Amaro 2004). The four climate groups encompassing distilleries are tropical [Aw], subtropical [(A)C], semi‐arid [BS] and temperate [C(w)]. If a variable affects microbial composition, samples in the cladogram should cluster according to that variable. We only observed clear grouping by the distillery to which the samples belong. ANOSIM results showed that only distillery is associated with fungal composition while distillery, fermentation stage, climate and region were associated with bacterial composition.

To identify the geographic, climatic and production variables that influence the microbial composition of the fermentation tanks, we analysed the beta diversity generating dendrograms based on the similarities of the microbiomes of the samples (Figure 3). The most important variables associated with each sample can be observed in Figure 3 as colour bars beside the dendrograms. The only grouping in which both fungal and bacterial ordination was statistically significant (ANOSIM) was according to the distillery (*r* = 0.5967, *p* = 0.0001 bacteria; *r* = 0.1703, *p* = 0.0315 fungi). This can also be appreciated at the dendrograms, where samples belonging to the same distillery often appear in the same clusters (Figure 3). No other variables were statistically significant for fungi, while grouping by fermentation stage, climate and region were also significant for bacteria (*r* = 0.2567, *p* = 0.006; *r* = 0.2768, *p* = 0.0001 and *r* = 0.1949, *p* = 0.0111, respectively).

Given the clustering observed by the distillery, we performed a Mantel test to evaluate if there is a correlation between geographic distance and microbial diversity of the fermentations. The resulting *r* value for bacteria was 0.2321 with a significance of 0.001, while for fungi they were 0.08247 and 0.101, respectively. For bacteria, these results showed that the microbial composition of two distilleries that are close by is more similar than the communities from distilleries that are further apart, which is in agreement with the ANOSIM results when assessing samples by distillery and region. In contrast, for fungi, the effect that we observed by distillery (ANOSIM) was not seen at all spatial scales, only at the level of distilleries. In summary, we observed that the distillery is an important determinant of both bacterial and fungal communities of the fermentation tanks, and, for bacteria, the fermentation phase, climate and producing region were also general determining factors.

### 3.5 Different Bacterial and Fungal Community Dynamics During *Agave* Fermentation

**FIGURE 4:**
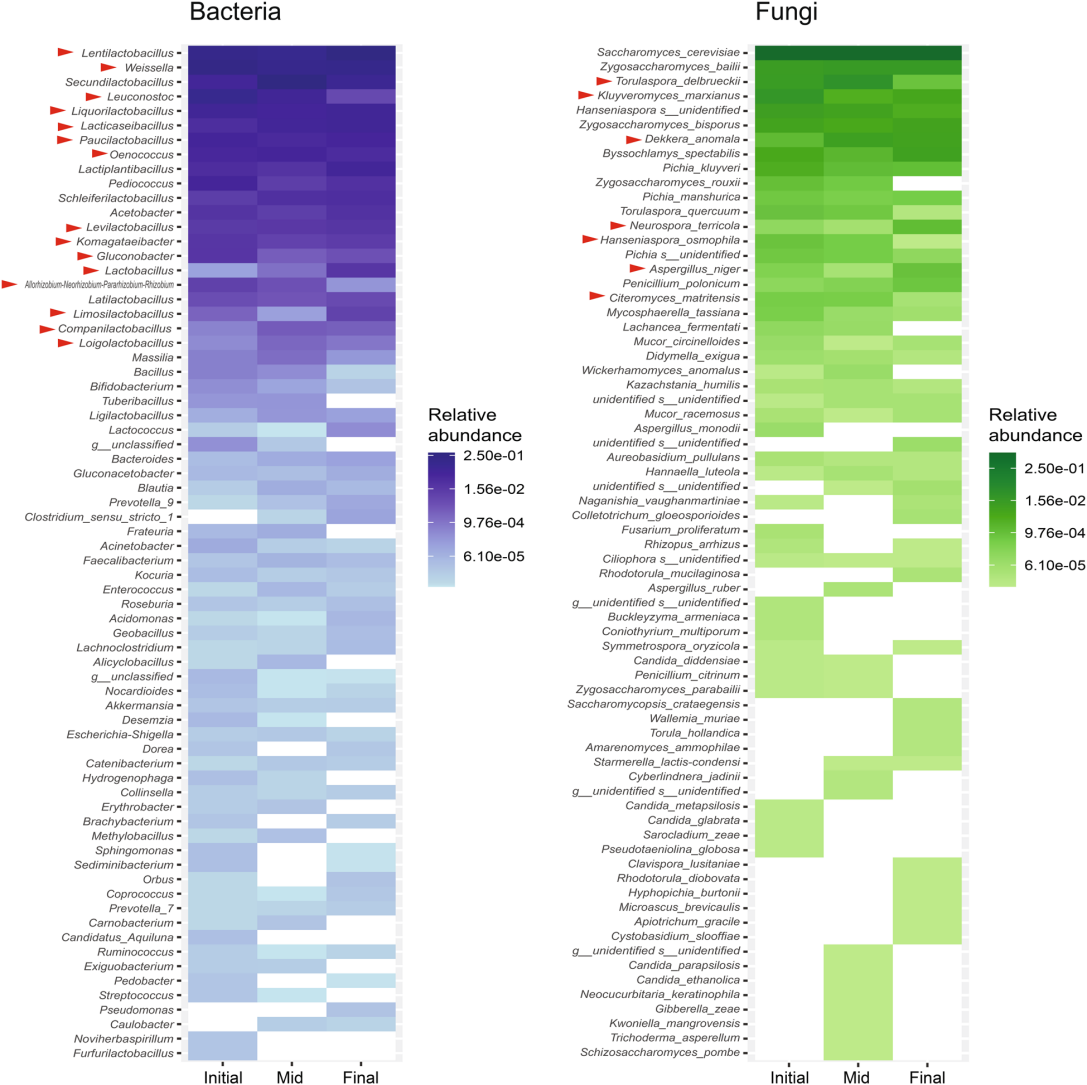
Microbial community changes through time during traditional agave fermentation. Relative abundance of the bacterial genera (left panel) and fungal species (right panel) were identified in each of the three fermentation phases in two distilleries that had fermentations of different times when sampled. Only the 100 most abundant taxa are shown ordered from top to bottom according to their overall relative abundance. Relative abundance is the number of reads of a genus/species relative to the total number of reads in the distillery or in all distilleries. Red arrowheads denote taxa that were identified to be differentially enriched between two fermentation stages by DESeq2 analysis as detailed in Figure S2.

Our general analyses of all distilleries revealed a significant association between the fermentation stage and the bacterial composition of agave fermentations (*r* = 0.2567, *p* = 0.006, ANOSIM). However, this was not observed for fungi, which was to some extent surprising given the ecological successions that have been observed in open fermentations employed for the production of other beverages (Pinto et al. 2015; Boynton and Greig 2016; Liu et al. 2020; Martiniuk et al. 2023). It is important to point out that the previous ANOSIM analysis was performed with the fermentation times of all sampled tanks. To investigate this further, we focused on two specific distilleries for which we had samples at different fermentation times (Figure 4). The samples of the two distilleries were grouped in three relative fermentation stages, each one representing a tercile of the total fermentation time (Initial, Mid and Final), and changes in diversity were evaluated through alpha diversity measures. The observed trends towards decreased diversity of both bacterial and fungal OTUs at the end of the fermentation were not statistically significant (Figures 4 and S1). However, for bacteria, the community structure was influenced greatly by the fermentation stage as reflected by the ANOSIM results when considering these two distilleries (*r* = 0.4608, *p* = 0.0128). In contrast, ANOSIM showed that the fungal communities are more uniform across the fermentation process (*r* = 0.02222, *p* = 0.4398) as had been suggested by the analysis of all fermentation samples.

To determine if there were specific bacterial and fungal taxa enriched at the different fermentation stages, we performed DESeq2 analysis. Although this method was developed to assess the differential expression of genes, it can be broadly used to estimate differences in count data from high‐throughput sequencing as has been done before for metabarcoding analysis (Halwachs et al. 2017). For bacteria, we only found enriched genera when comparing the initial or mid stages with the final one, and there were more than double the number of genera enriched in the initial or mid stages (Figure S2). *Allorhizobium*, *Komagataeibacter*, *Lentilactobacillus*, *Leuconostoc*, *Levilactobacillus*, *Liquorilactobacillus*, *Oenococcus* and *Paucilactobacillu*s were enriched in both the initial and mid‐fermentation phases. The first two genera belong to the Proteobacteria and the rest are Firmicutes. *Companilactobacillus, Lactobacillus* and *Limosilactobacillus*, all Firmicutes, were enriched in the final stage compared to the two other stages. All the differentially enriched genera are part of the core bacterial component of agave fermentations.

In the case of fungi, there were only enriched species when comparing the initial and final fermentation stages with the mid phase (Figure S2). *K. marxianus* was enriched at the beginning of the fermentation when compared to the mid stage, while *Dekkera anomala* showed the inverse enrichment. *Citeromyces matritensis, Hanseniaspora osmophila* and *T. delbrueckii* were enriched in the mid‐fermentation phase when compared to the final stage, while *Aspergillus niger* and *Neurospora terricola* showed the reverse pattern. All these species are ascomycetes, and *K. marxianus* and *T. delbrueckii* belong to the core fungal component of agave fermentations.

The statistically significant association between bacterial composition and fermentation stage suggested the possible occurrence of an ecological succession throughout agave fermentation, aligning with findings from other fermentation processes (Pinto et al. 2015; Boynton and Greig 2016; Liu et al. 2020; Martiniuk et al. 2023). However, in agave fermentations, the changes in fungal composition may be more subtle compared to other systems. Therefore, further analyses with increased sample size and specifically tailored to compare different stages are probably needed to detect such subtle variations in the fungal composition of these fermentations.

### 3.6 Specific Bacterial Genera Are Enriched in Fermentations of Certain Agaves Within a Distillery

Although clustering by the agave species used in the fermentation was not statistically significant when considering all the samples, we included a distillery where fermentations of different agaves were being carried out in parallel (D13). This offered a unique opportunity to assess the influence of the plant employed as a substrate because all other variables were similar between the tanks. As can be observed in the dendrograms of Figure 3, the bacterial microbiomes of the samples of *Agave potatorum* from this distillery clustered together while the ones of other agaves, including several of *A. angustifolia* , did not. We did not observe clear clustering by agave species for the fungal microbiomes of this distillery. Performing a DESeq2 analysis, we identified 16 bacterial OTUs that were differentially enriched between the microbiomes of the *A. potatorum* samples and the rest of the agaves (Figure S3). All of them but one are Firmicutes, and several belong to the core bacterial microbiome. Importantly, when the same analysis was performed, but instead of comparing the *A. angustifolia* fermentations against the rest of the samples, only one OTU was differentially enriched. Although further experiments are needed to better understand the influence of the agave species on the microbiome, our findings suggest that the specific biochemical composition of the plants could have determining effects on the microbial composition of agave fermentations, at least in the case of *A. potatorum*.

### 3.7 The Microbiome of Agave Spirit Fermentations and Pulque Are Considerably Different

Pulque is a traditional Mexican beverage produced from the fermentation of agave sap. Contrary to what is done to produce agave spirits, agave sap is not cooked for pulque production, and the beverage is the direct ferment without distillation. The microbiome involved in pulque fermentations has been well characterised using metagenomic approaches (Rocha‐Arriaga et al. 2020; Astudillo‐Melgar et al. 2023) and given the similarity in fermentation substrate, agave sap vs. cooked agave must, it is an excellent point of comparison to better understand the microbial communities involved in the production of agave spirits. For the analysis of pulque, we used 1,399,910 previously sequenced 16S and ITS pair‐end reads (Rocha‐Arriaga et al. 2020). These sequences were generated from six pulque samples with a mean of 74,882.16 ± 336.61 reads per sample for bacteria and 10,782.16 ± 280.71 reads for fungi. On average, the bacterial communities of pulque samples had a higher number of observed OTUs (3105.5 ± 277.09, Table S3) than samples from agave spirits (1002.08 ± 316.47). In agreement, the bacterial communities from agave spirit fermentations are less diverse than pulque, displaying a Shannon index (H′) of 3.26 ± 0.66, while the community of pulque showed 3.64 ± 0.51. Species dominance, evaluated through the Simpson index, presented similar values in agave spirit fermentations (0.86 ± 0.12) than in pulque samples (D = 0.85 ± 0.06). Similarly, the pulque microbiomes on average showed a higher amount of observed ITS OTUs (114.33 ± 98.82) than the ones from agave spirit samples (92.85 ± 39.5). For fungi, both Shannon and Simpson indices were also more elevated for the communities of pulque samples (H′ = 2.18 ± 0.21; *D* = 0.80 ± 0.03 in pulque, against H′ = 1.20 ± 0.74; 0.44 ± 0.27 in agave spirits).

**FIGURE 5:**
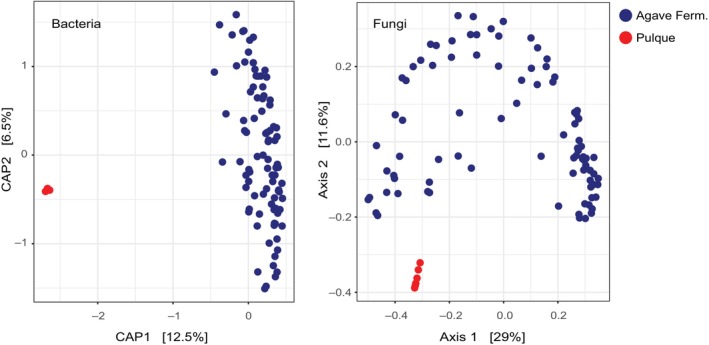
The microbiomes of pulque and agave spirit fermentations are considerably different. Beta diversity analysis of bacterial (left panel) and fungal communities (right panel) in pulque (red dots) and fermentations used for agave spirit production (blue dots). For bacteria, we used PCA with an unweighted UniFrac distance matrix, and for fungi, we used PcoA based on a Jaccard similarity matrix.

Overall, a total of 100 bacterial genera and 49 fungal species were shared between pulque and agave spirit samples. Despite the communalities, the microbiomes of the two types of fermentations are clearly different. Agave spirit fermentations have 788 bacterial genera and 282 fungal species that are exclusive to these fermentations, while pulque fermentations have only 9 and 12, respectively (Tables S2 and S3). Furthermore, both bacterial and fungal beta diversity analyses by multivariate ordination using CAP showed evident separation of pulque and agave spirit microbiomes (Figure 5). Overall, each bacterial and fungal ordination explains 19% and 40% of the observed variance for bacteria and fungi, respectively, and showed that the communities from agave spirit fermentations differ more than the pulque samples (Figure 5). In addition to the taxa that were only found in one type of fermentation, DESeq2 analysis identified a total of 31 bacterial genera that were present in both environments but were overrepresented in agave spirit fermentations, and only eight that were overrepresented in pulque. Similarly, 12 species of fungi were significantly enriched in agave spirit fermentations, while eight species were overrepresented in pulque. In sum, our comparison showed important differences between the microbiomes of agave spirit fermentations and pulque even though both employ agave as the raw material.

## 4 Discussion

### 4.1 A Country‐Wide Analysis of Microbial Diversity in Traditional Agave Fermentations

The production of agave spirits is culturally and commercially of central importance to Mexico. In recent decades, the aromas and flavours of these beverages have been increasingly appreciated worldwide, to the extent that other countries such as South Africa, Australia and Kenya have also started producing agave spirits (Smith 2017; Yan et al. 2020). Most of the focus on this activity has been placed on the plant variety and distillation practices employed, leaving aside the microorganisms that perform the fermentation (Arellano‐Plaza et al. 2022). For example, the official Mexican standards of the *mezcal* designation of origin (NOM‐070‐SCFI‐2016) specify the geographical location and developmental stage and the way agave plants are to be transported, but in terms of the fermentation it only mentions a wide variety of possible vat materials. The microorganisms that perform the fermentation are largely unknown for traditional distilleries in which no inocula are employed and the process is presumably performed by autochthonous bacteria and yeasts from the surrounding environment. To better understand these microbial communities, here we characterised the fungal and prokaryotic composition of a country‐wide collection of fermentation musts employing a metabarcoding approach. Despite the wide range of geo‐climatic characteristics and production practices encompassed by the distilleries, we did observe a core set of bacterial genera and fungal species that are prevalent in most fermentations (Table 1). Lactic acid bacteria were the most common, although we also identified a considerable number of acetic acid bacteria. In the case of fungi, Ascomycetes were by far the most ubiquitous and abundant since other phyla represented less than 10% of the total number of OTUs. These findings are in agreement with the microorganism that had been isolated in previous studies from this type of fermentations, which as a whole covered close to 15 distilleries (Colón‐González et al. 2024). Importantly, our study revealed extensive intra‐generic diversity among prokaryotes and intra‐specific variability among fungi, with the most abundant genera and species encompassing multiple OTUs.

Our work considerably expanded the list of microorganisms associated with traditional agave fermentations. This is especially true for prokaryotes that had been barely considered before—close to 98% of the bacterial genera that we identified had not been described in this environment. For fungi, over 90% of the genera and species detected had also not been isolated from agave fermentations. Several of the taxa that we considered here as the core of the microbiomes (Table 1) had not been isolated in previous work, showing the importance of our effort to better understand these communities. For prokaryotes, this could be expected since only a few previous studies focused on them (Escalante‐Minakata et al. 2008; Narvaez‐Zapata et al. 2010; Kirchmayr et al. 2017). In the case of fungi, it was surprising to find *P. polonicum* , *M. tassiana* and *A. pullulans* as prevalent taxa in agave fermentations. All these species are known to be widespread in a variety of environments and *P. polonicum* and *A. pullulans* have been used for biotechnological applications (Chi et al. 2009; Ding et al. 2013). On the other hand, *P. polonicum* is usually associated with food spoilage and *M. tassiana* belongs to a large genus of phytopathogenic fungi (Barr 1958; Duduk, Vasic, and Vico 2014). It will be interesting to define whether the three species contribute positively or negatively to the fermentation dynamics and organoleptic properties in agave spirit production.

We did miss bacterial and fungal genera that had been identified by previous studies. This agrees with our diversity estimations which suggested that a fraction of the microbial communities in each distillery was still to be determined. The previously identified microorganisms not found in the present study are most probably rarer species with focalised distribution since they had not been reported in more than one study. Changes in the taxonomy of microorganisms also explained some of the discrepancies with previous reports as was the case for *Zymomonas*. Comparing the microbial diversity of agave fermentations with that of other fermented foods and beverages, the bacterial diversity (Shannon index) is close to average diversity in other fermentations, while fungal diversity is closer to the least diverse environments such as wine fermentations (Leech et al. 2020; Rocha‐Arriaga et al. 2020; Kharnaior and Tamang 2023; Martiniuk et al. 2023; Qi, Bruni, and Klasson 2023). Overall, our work sets the basis for a more comprehensive understanding of the composition and diversity of the microbial communities responsible for the fermentation needed to produce traditional agave spirits.

### 4.2 Composition of Microbial Communities in Agave Fermentations Is Defined at the Level of Distillery

As in other open fermentation systems, it is expected that the climatic factors and elaboration practices will determine the microbial composition of agave fermentations at different geographical scales. For example, fermentations within a producing region would be expected to have microbiomes that are more similar to each other than to those of a different region. Our work offered the first opportunity to investigate such effects throughout the country as previous efforts had mainly focused on one or two distilleries and employed isolation methods that made it difficult to directly compare across studies. Differences in the microbiomes were observed for bacteria and fungi only when compared at the distillery level. This suggests that the microbiome of agave fermentations is firstly defined locally by the specific characteristics and practices of each production site. The few differences that we found in the microbiomes of tanks of distinct fermentation stages and agave plants within single distilleries are in agreement with this suggestion; the conditions within the distillery are more determinant than other factors.

Our results also showed that the fungal and bacterial components of agave fermentations are differentially influenced by environmental factors. Apart from the observations at the distillery level, significant differences were observed in the bacterial microbiomes of samples of distinct fermentation stages, climates and producing regions, but not in the fungal component of agave fermentations. Overall, we observed less diversity in the fungal microbiome with 13 species that form a core fungal component that is present in almost all fermentations sampled throughout the country (Table 1), and these species showed considerable intra‐specific diversity. It remains to be seen whether fungal diversity within species is better correlated with geographical distribution.

Our findings are to some extent contrasting to what has been observed for microbiomes of wine fermentations, specifically for the fungal microbiome. The bacterial and fungal communities of wineries in California and Portugal, for example, have been reported to be specific to wine appellations, especially at early stages of fermentation (Pinto et al. 2015; Bokulich et al. 2016). It is worth pointing out that the wine regions analysed are considerably smaller than the area where the agave fermentations sampled in this study are located and that endogenous microorganisms from the plant are not eliminated through a cooking step. Differences between the microbiomes of individual vineyards have also been found, similar to what we observed between agave distilleries, but in addition, there were differences between viticultural regions. These differences have been suggested to contribute to the specific characteristics of the wines from the above‐mentioned appellations. The differences in chemical composition between agave and grape musts may also contribute to the dissimilarities in microbial patterns between the two fermentation environments. For example, the range of ethanol content is narrower in agave fermentations since the final concentration only gets to about 6% (Colón‐González et al. 2024) while in wine it is often higher than 10%.

Microbiomes of the fermentations used in the production of agave spirits may more closely resemble those involved in miso fermentation in Japan. A recent study revealed that latitude has a relatively small impact on the fungal and bacterial communities of these fermentations, while the effect of the brewery was strong even when fungi were inoculated by the producers (Koide, Kanauchi, and Hashimoto 2024). Efforts to standardise miso production employing specific fungal inoculants overlook the resilience of unique brewery‐specific microbiomes. Similarly, attempts to standardise agave fermentations may encounter obstacles due to the diverse microbial communities shaped by the local distillery conditions and practices.

The wide range of geographical and climatic characteristics encompassed by the production sites of agave spirits added to the great variety of cultural differences in production practices make agave fermentations a very diverse environment. Therefore, it is possible that a larger number of distilleries and fermentation tanks needs to be analysed to better identify the factors that define the microbial communities of agave fermentations. Yet, our work represents the most comprehensive effort to date to understand these microbiomes and provides valuable insights regarding the specific taxa that constitute them. In addition, the collection of agave musts that we generated offers the opportunity for future detailed chemical characterisation of the ferments, which could reveal other factors that are associated with the microbiome.

### 4.3 Fermentation Time, Agave Species and Production Practices Influence the Microbiomes of Agave Fermentations

We observed a trend towards decreased alpha diversity of both bacterial and fungal OTUs towards the end of fermentation, even if the differences were not statistically significant (Figure S1). The ANOSIM test revealed a significant influence of the fermentation stage on the structure of the prokaryotic communities in agave fermentations, highlighting a clear differentiation in bacterial beta diversity across stages. In addition, specific bacterial groups were enriched at different fermentation stages. The observed decrease in diversity towards the end of fermentation and the significant correlation between bacterial diversity and fermentation stage are consistent with findings from other fermentation processes (Pinto et al. 2015; Bokulich et al. 2016). In contrast, fungal communities appeared to be more uniform throughout the fermentations. In general, our results are in accordance with the survey of yeast diversity in traditional agave fermentations that we recently carried out employing culture‐based methods (Gallegos‐Casillas et al. 2023). This work centred in yeast communities revealed composition changes through time, even if the alpha diversity was not statistically different across stages. However, in contrast with the metabarcoding results presented here, the previous analyses of yeast beta diversity did show a statistically significant decrease as a function of the fermentation phase, even when the magnitude of the change was small (Gallegos‐Casillas et al. 2023).

The observed decrease in diversity towards the end of fermentation and the statistically significant correlation between bacterial diversity and fermentation stage align with findings from other alcoholic fermentation processes (Costa et al. 2015; Pinto et al. 2015; Huang et al. 2023). However, in agave fermentations, the changes in fungal composition may be more subtle compared to other fermentations (Gallegos‐Casillas et al. 2023). To gain a more comprehensive understanding of the ecological succession in spontaneous agave fermentations, further analyses with increased sample size and specifically tailored to compare different stages are necessary. These additional investigations will uncover finer details of microbial dynamics and shed light on the intricate ecological processes occurring during agave fermentation.

The plant species used to produce agave spirits are thought to be one of the most influential factors contributing to the flavour and aroma of these beverages. There are spirits made from agave varieties, such as *A. potatorum*, that are highly valued by consumers and their prices are considerably higher. It is possible that some of the attributes contributed by the specific agave variety may come indirectly from the influence that the plant components have on the microbial community responsible for the fermentation. By analysing fermentations of different agave plants within the same distillery, we did observe specific bacterial genera associated with fermentations of *A. potatorum*. Clearly, further work is needed including sampling strategies specifically designed to better understand this association, but our findings suggest the possibility that the effects of the plant variety on the organoleptic properties of the spirits may occur by determining the composition of the microbiomes of the fermentations.

Production practices are another determinant of the quality of agave spirits. For example, distillation in clay pots is used in some breweries because it is thought to add specific flavours, despite the fact that it is a less efficient distillation system. In terms of a practice that could affect the fermentation process, we did not observe any statistically significant differences in the microbial communities when samples were grouped by the material of the fermentation tank and the grinding method used. As for other variables not found to be associated with the microbial composition, it is possible that a larger set of samples is needed to detect such effects given the complexity and diversity of the system. We did observe an important difference between the bacteria and fungi of pulque and agave spirit fermentations. At a fundamental level, the raw material used to produce both beverages are the oligosaccharides of agave plants. However, in the production of agave spirits, compounds from the plant have been subject to high temperatures for considerable periods of time. Apart from breaking oligosaccharides and chemically transforming other components of the plant, this step presumably eliminates all microorganisms from the plant and its surroundings and inoculation of agave must by microorganisms presumably starts after cooking when steams cool down. A previous study suggested that tools in the distillery could serve as yeast reservoirs for each new fermentation (Lachance 1995), but further work is needed to understand the ecological dynamics of these microorganisms. Our findings suggested that cooking the agave steams has important implications for the composition of the microbial communities of agave fermentations.

### 4.4 Concluding Remarks

To the best of our knowledge, the work presented here represents the most comprehensive analysis of the bacterial and fungal communities involved in the open fermentations needed to produce traditional agave spirits. We identified hundreds of species that had not been associated with this environment before and detected local differences in the microbiomes of the sampled distilleries. The local differences suggest that the sum of historical and environmental factors and the production practices of each distillery determine the microbial composition of agave fermentations. In turn, the specific microbiome of each distillery may contribute to the properties of the corresponding agave spirit, adding to the *terroir* of these beverages. Given the rapid pace at which the production of agave spirits is changing due to the increasing demand, the microbial communities here identified will serve as the basis to better understand and preserve the fermentations in this unique traditional process.

## Author Contributions

**Angélica Jara‐Servin:** methodology, investigation, writing – original draft, formal analysis, visualization. **Luis D. Alcaraz:** methodology, writing – review and editing, supervision, formal analysis, visualization. **Sabino I. Juarez‐Serrano:** methodology, investigation. **Aarón Espinosa‐Jaime:** methodology, investigation. **Ivan Barajas:** methodology, investigation. **Lucia Morales:** conceptualization, funding acquisition, writing – review and editing. **Alexander DeLuna:** conceptualization, writing – review and editing, funding acquisition. **Antonio Hernández‐López:** conceptualization, funding acquisition, writing – review and editing, supervision. **Eugenio Mancera:** conceptualization, formal analysis, writing – original draft, funding acquisition, supervision, visualization, investigation.

## Conflicts of Interest

The authors declare no conflicts of interest.

## Supporting information


**Table S1.** Characteristics of the fermentation tanks from where the samples were collected.

**Table S2.** Bacterial and fungal taxa identified in traditional agave fermentations. This table contains four tabs: two for prokaryotes (16S) and two for fungi (ITS). For each type of microorganism, there is a list of all the OTUs identified (suffix “_OTU”) and of all the genera/species identified (suffixes “_Genus” and “_Species”). At the OTU tabs, for each sample, the first four rows provide the total number of raw reads (“Raw_paired_sequences”), the total number of assembled reads (“Assembled_sequences”), the total number of assembled and filtered reads (“Filtered_sequences”) and the total number of OTUs (“Total_OTUs”).

**Table S3.** Bacterial and fungal OTUs and taxa identified in pulque.

**Figure S1.** Diversity analysis of the different fermentation stages in two distilleries. Diversity estimators of the three fermentation stages analysed in two specific distilleries that had tanks of different times when sampling took place.

**Figure S2.** DESeq2 analyses of the different fermentation stages in two distilleries. DESeq2 results of the comparison of the three different fermentation stages at the level of OTU of the same two distilleries. Only comparisons where there were differentially enriched OTUs are shown.

**Figure S3.** DESeq2 analysis in different agave species within a single distillery.

Data S1.

## Data Availability

The data that support the findings of this study are openly available in NCBI at https://www.ncbi.nlm.nih.gov/, under BioProject PRJNA1085712.
