# Supplementary figures and images for: Microbial Communities in Agave Fermentations Vary by Local Biogeographic Regions
Source: Environ Microbiol Rep. 2025 Jan 24;17(1):e70057. doi: 10.1111/1758-2229.70057 (PMC11761429; doi:10.1111/1758-2229.70057)

Supplementary Figure 1

Bacteria

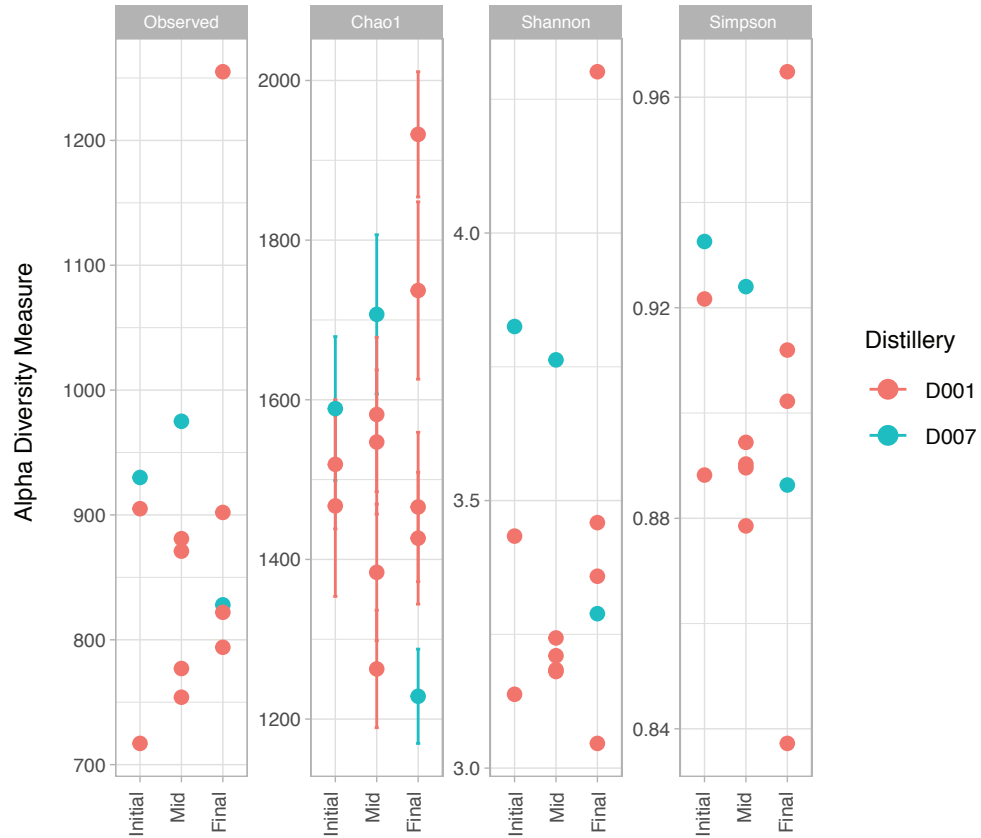

Fungi

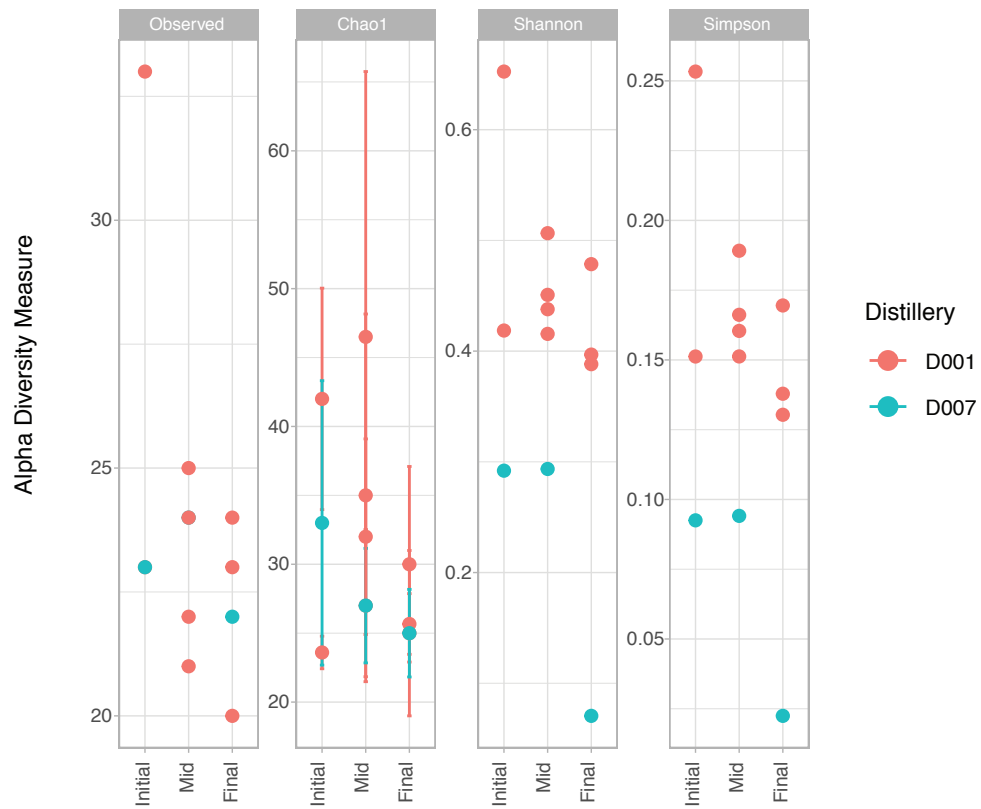

Supplement: Supplementary file 4 — Figure S1. Diversity analysis of the different fermentation stages in two distilleries. Diversity estimators of the three fermentation stages analysed in two specific distilleries that had tanks of different times when sampling took place. [file EMI4-17-e70057-s005.pdf]

Supplementary Figure 3

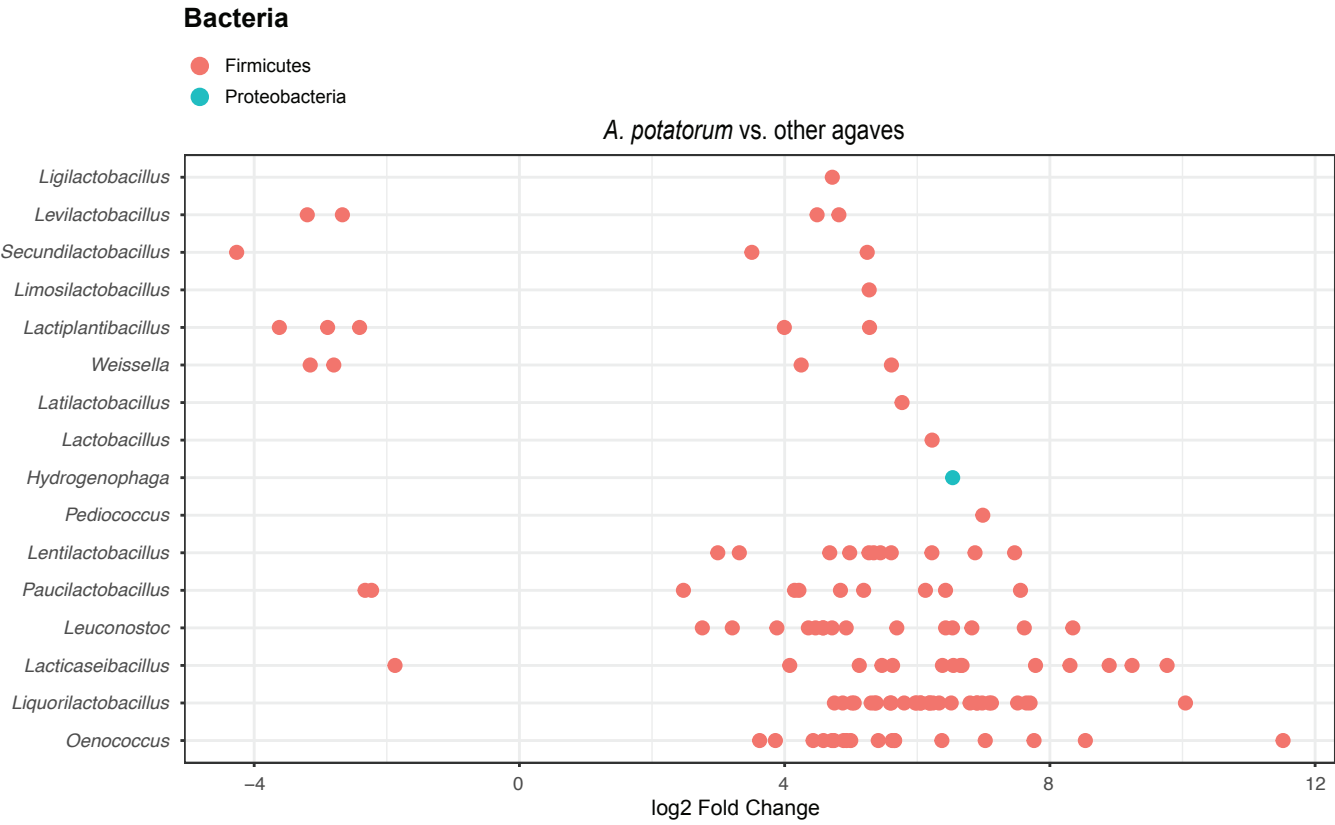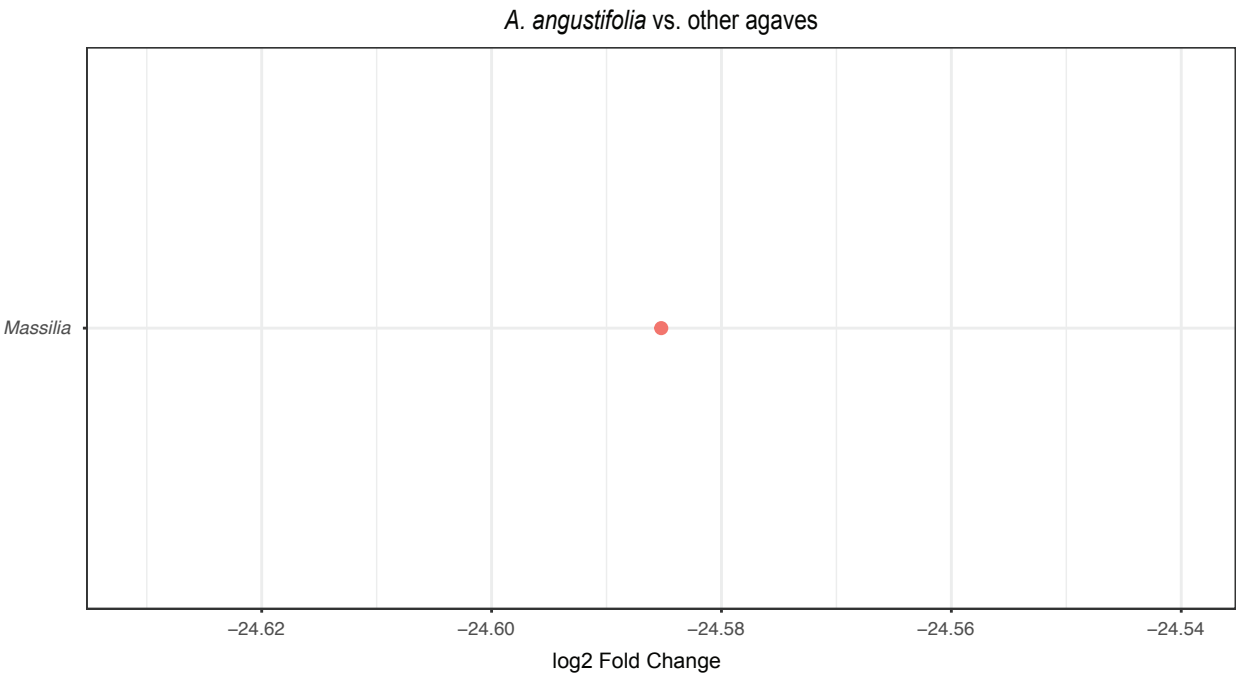

Supplement: Supplementary file 6 — Figure S3. DESeq2 analysis in different agave species within a single distillery. [file EMI4-17-e70057-s002.pdf]
