# Supplementary material for: Microbial Communities in Agave Fermentations Vary by Local Biogeographic Regions
Source: Environ Microbiol Rep. 2025 Jan 24;17(1):e70057. doi: 10.1111/1758-2229.70057 (PMC11761429; doi:10.1111/1758-2229.70057)

Supplementary Figure 2

Bacteria

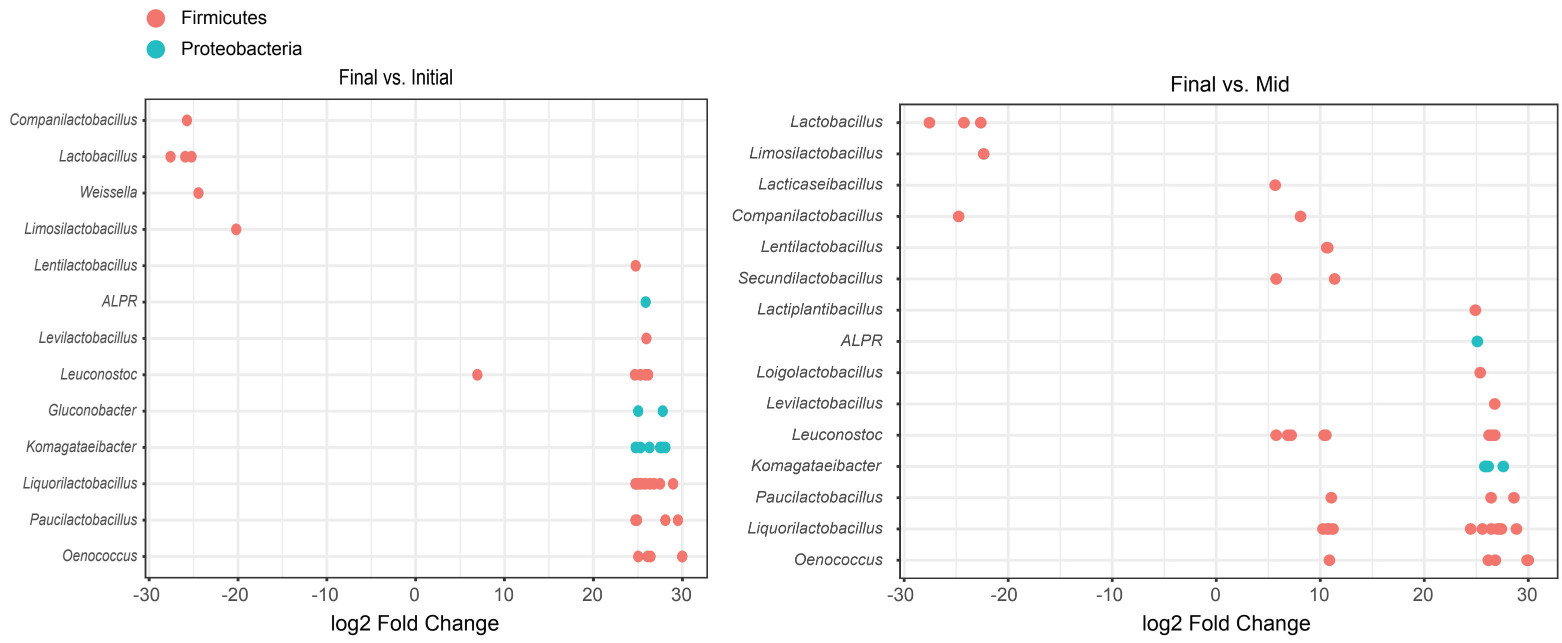

Fungi

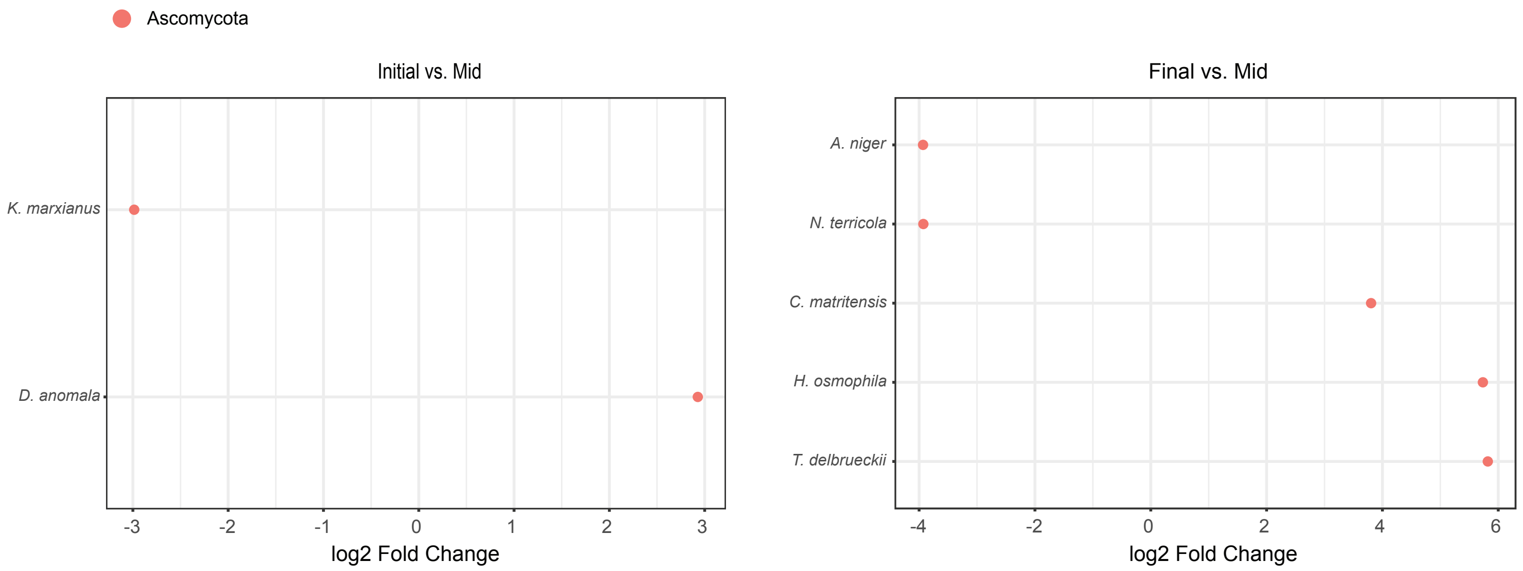

Supplement: Supplementary file 5 — Figure S2. DESeq2 analyses of the different fermentation stages in two distilleries. DESeq2 results of the comparison of the three different fermentation stages at the level of OTU of the same two distilleries. Only comparisons where there were differentially enriched OTUs are shown. [file EMI4-17-e70057-s007.pdf]
