# Supplementary material for: Microbial Communities in Agave Fermentations Vary by Local Biogeographic Regions
Source: Environ Microbiol Rep. 2025 Jan 24;17(1):e70057. doi: 10.1111/1758-2229.70057 (PMC11761429; doi:10.1111/1758-2229.70057)
Supplement: Supplementary file 7 — Data S1. [file EMI4-17-e70057-s004.pdf]

Las comunidades microbianas asociadas a las fermentaciones de agave son influenciadas localmente a través de diversas regiones biogeográficas

Angélica Jara-Servin[1#], Luis D. Alcaraz[1#], Sabino I. Juarez-Serrano[2], Aarón Espinosa-Jaime[3], Ivan Barajas[3], Lucia Morales[4], Alexander DeLuna[5], Antonio Hernández-López[3*] and Eugenio Mancera[2*]

[1]Laboratorio de Genómica Ambiental, Departamento de Biología Celular, Facultad de Ciencias, Universidad Nacional Autónoma de México.

[2]Departamento de Ingeniería Genética, Centro de Investigación y de Estudios Avanzados del Instituto Politécnico Nacional, Unidad Irapuato, Irapuato, Mexico

[3]Escuela Nacional de Estudios Superiores Unidad León, Universidad Nacional Autónoma de México, Guanajuato, México

[4]Laboratorio Internacional de Investigación sobre el Genoma Humano, Universidad Nacional Autónoma de México, Querétaro, Mexico

[5]Unidad de Genómica Avanzada, Centro de Investigación y de Estudios Avanzados del Instituto Politécnico Nacional, Irapuato, Mexico

# Equal contribution

*Correspondenia: Antonio Hernández-López (ahernandez@enes.unam.mx) and Eugenio Mancera (eugenio.mancera@cinvestav.mx)

Keywords: agave fermentation, microbial communities, metabarcoding, open fermentation

RESUMEN

La producción de destilados tradicionales de agave en México, como el mezcal, es un proceso ancestral que depende de microorganismos ambientales para fermentar el mosto cocido de las plantas de agave. El análisis de estos microorganismos permite comprender la dinámica de las comunidades microbianas en la interfaz entre entornos naturales y humanos. Para conocer estas comunidades, en este estudio, realizamos secuenciación de amplicones 16S e ITS de 99 tanques de fermentación en 42 destilerías en México. Las especies de agave utilizadas, las prácticas de producción, las condiciones climáticas y las características biogeográficas variaron considerablemente entre los sitios. Sin embargo, encontramos taxones presentes en la mayoría de las fermentaciones, lo que sugiere un núcleo microbiano característico de estas comunidades. La única variable consistentemente asociada con la composición de las comunidades bacterianas y fúngicas fue la destilería, lo que indica que los microbiomas están determinados por prácticas de producción locales y características biogeográficas únicas de cada sitio. La etapa de fermentación, el clima y la región productora también se asociaron con la composición de la comunidad, pero solo para procariontes. Además, el análisis de varios tanques dentro de tres destilerías reveló taxones que se enriquecieron en etapas específicas de fermentación o especie de agave. Este estudio proporciona un análisis exhaustivo del microbioma de las fermentaciones de agave, contribuyendo conocimientos clave para su manejo y conservación.

1 INTRODUCCIÓN

México se destaca por su diversidad biológica y cultural, resultado de su compleja historia geológica y social. Por ejemplo, cuenta con uno de los mayores números de especies de reptiles, robles y pinos del mundo (Farjon 1996, Nixon 2006, Suazo-Ortuño et al. 2023). Además, es una de las regiones más multilingües, y su población es predominantemente mestiza (Eberhard et al. 2020, Sohail et al. 2023). Sin embargo, sabemos relativamente poco sobre su diversidad microbiana en hábitats tanto naturales como humanos. La producción de bebidas destiladas a partir de plantas de agave es una actividad profundamente arraigada en México. No está claro si esta práctica data de tiempos precolombinos (Machuca Chávez 2018, Ojeda-Linares et al. 2021), pero actualmente se realiza desde las provincias más meridionales de México hasta los estados fronterizos del norte con Estados Unidos (Serra Puche y Lazcano Arce 2016, Cabrera-Toledo et al. 2022). Su amplia distribución probablemente está relacionada con la abundancia de plantas de agave en todo el territorio mexicano, siendo esta región el centro de diversidad del género (Trejo et al. 2018).

El tequila es posiblemente el destilado de agave más conocido y comercial, producido en entornos altamente industrializados. Sin embargo, la mayoría de los otros destilados de agave, como el mezcal, la raicilla o el bacanora, se producen de forma más artesanal, caracterizada por fermentaciones abiertas y "espontáneas" en las que los productores no dependen de un inóculo microbiano establecido (Arellano-Plaza et al. 2022). En cambio, los corazones de agave cocidos y macerados se fermentan por microorganismos provenientes del entorno circundante. Por lo tanto, la caracterización del microbioma en las fermentaciones de agave no solo es esencial para

comprender mejor la producción de estas bebidas de importancia tradicional y comercial, sino que también brinda información sobre las comunidades microbianas naturales de México.

La enorme área donde se producen las bebidas espirituosas de agave — una región de mayor tamaño que Europa Occidental — presenta una amplia variedad de condiciones ambientales y prácticas de producción (Arellano-Plaza et al. 2022). Por ejemplo, existen destilerías situadas casi al nivel del mar, mientras que otras se encuentran a 2,000 metros de altitud, abarcando climas áridos, semiáridos y subhúmedos en áreas subtropicales y tropicales. Esto da lugar a un amplio rango de temperaturas y precipitaciones anuales que determinan, entre otras cosas, las especies de agave que pueden crecer en cada región. Se han reportado más de 50 especies diferentes de agave utilizadas para la producción de bebidas espirituosas de agave, y sus contenidos de azúcar, nitrógeno y otros metabolitos puede variar entre ellas (Colunga-GarciaMarin et al. 2017). El proceso de cocción hidroliza los fructanos, los carbohidratos más abundantes en las plantas de agave, liberando azúcares simples, como la fructosa, para la etapa de fermentación (se han reportado valores de azúcar de 20 g/L a 295 g/L, (Colón-González et al. 2024). Sin embargo, la cocción también produce compuestos que pueden inhibir el crecimiento de microorganismos, como el 5-hidroximetilfurfural y el furfural (Mancilla-Margalli y López 2002). Junto con metabolitos de las plantas de agave, como las saponinas, estos compuestos pueden hacer de las fermentaciones de agave un entorno desafiante para los microorganismos. Para una revisión en profundidad de las fermentaciones de agave como un ambiente ecológico para microorganismos, véase (Colón-González et al. 2024).

Los microorganismos más abundantes identificados en las fermentaciones de agave mediante métodos microbiológicos basados en cultivo, con sus sesgos intrínsecos, son levaduras ascomicetas y bacterias de ácido láctico (Escalante-Minakata et al. 2008, Kirchmayr et al. 2017, Gallegos-Casillas et al. 2023, Colón-González et al. 2024). La mayoría de los estudios previos se han centrado principalmente en las levaduras, ya que se considera que son los principales contribuyentes a la producción de etanol. La caracterización más extensa hasta la fecha de las comunidades fúngicas en fermentaciones tradicionales de agave reveló recientemente un núcleo de seis levaduras ascomicetas que se aislaron frecuentemente en estas fermentaciones (Gallegos-Casillas et al. 2023). Estas seis especies también habían sido aisladas en estudios anteriores, incluso cuando solo se realizaron en unas pocas fábricas, en su mayoría ubicadas en el estado de Oaxaca, principal productor de mezcal (Kirchmayr et al. 2017, Nolasco-Cancino et al. 2018). Se considera que las diferentes especies de levadura involucradas en estas fermentaciones no solo contribuyen a la producción de etanol, sino también a la síntesis de otros compuestos con propiedades organolépticas.

Se sabe mucho menos sobre el microbioma procariota en las fermentaciones de agave, y las bacterias en otras fermentaciones antropogénicas a menudo se consideran contaminantes que pueden provocar deterioro. Los cuatro estudios previos que analizaron procariotas en fermentaciones tradicionales para la producción de bebidas destiladas de agave identificaron principalmente especies de bacterias ácido-lácticas, pero también bacterias acéticas y bacterias formadoras de esporas (Escalante-Minakata et al. 2008, Narvaez-Zapata et al. 2010, Kirchmayr et al. 2017, Kirchmayr et al. 2024). Las capacidades de producción de etanol de algunas de estas especies sugieren que las bacterias podrían tener un papel más importante en la producción de

destilados tradicionales de agave de lo que se pensaba anteriormente (Kirchmayr et al. 2017). A pesar de los esfuerzos previos, que se basaron principalmente en métodos microbiológicos de cultivo, aún existen muchas preguntas fundamentales sin respuesta sobre el microbioma de las fermentaciones tradicionales de agave. De hecho, no está claro si las especies de levaduras aisladas son realmente los hongos predominantes en estas fermentaciones. Es posible que existan otros hongos abundantes que no se aíslan comúnmente mediante métodos microbiológicos clásicos. También se desconoce qué tipos de bacterias juegan un papel en las diferentes regiones donde se producen las bebidas de agave.

Para obtener una visión más profunda de los microbiomas bacteriano y fúngico en las fermentaciones de agave, realizamos la secuenciación de amplicones del gen 16S rRNA (16S) y del espacio intergénico transcrito interno ribosomal (ITS) a partir de muestras de mosto recolectadas en 99 tanques de fermentación de 42 destilerías en las regiones productoras de bebidas de agave en México. Nuestros resultados mostraron que, a pesar de la diversidad en las prácticas de producción y los parámetros biogeográficos, existe un núcleo de especies que define las comunidades microbianas en todo el país. Muchos de los taxones coinciden con especies previamente aisladas de estas fermentaciones, pero también identificamos especies prevalentes que nunca antes se habían asociado con fermentaciones de agave. Aunque los Ascomycota fueron, de lejos, los hongos más comunes, también identificamos hongos de otros cinco filos, mostrando una considerable diversidad fúngica general. El microbioma bacteriano es aún más diverso que el componente fúngico, y está dominado por especies de bacterias ácido-lácticas. La única asociación en la composición del microbioma que observamos tanto en bacterias como en hongos fue con la destilería a la que pertenecían las muestras. Hasta donde sabemos, nuestro trabajo representa el

primer análisis a nivel nacional que emplea enfoques de metabarcoding del microbioma en fermentaciones utilizadas para la producción tradicional de bebidas espirituosas de agave, ampliando considerablemente nuestro entendimiento de estas comunidades.

2 MATERIALES Y MÉTODOS

2.1 Recolección de muestras de fermentación de agave, extracción de ADN y secuenciación

Las muestras de fermentación de agave fueron recolectadas como parte del trabajo de campo realizado por el consorcio YeastGenomesMx en las siete regiones donde se producen destilados de agave en México (Figura 1). Para cada muestra, se tomaron 4 ml de mosto de agave con una pipeta serológica estéril, y se almacenaron en viales criogénicos para su congelación inmediata en nitrógeno líquido (Gallegos-Casillas et al. 2023). En varias destilerías, se muestrearon más de un tanque de fermentación. Los metadatos asociados a cada muestra se recolectó en el campo según el método descrito previamente (Gallegos-Casillas et al. 2023). En el laboratorio, las muestras se almacenaron a -80 °C hasta su procesamiento. El ADN total se extrajo utilizando el kit ZymoBIOMICS DNA Miniprep (D4300) de Zymo Research. Las extracciones se realizaron a partir de 500 µl de muestra siguiendo el protocolo del fabricante, con una incubación adicional de 15 minutos a 70 °C después de la lisis y antes de la centrifugación y filtración. La cantidad, pureza e integridad del ADN se monitorearon con un Nanodrop, fluorometría en un Qubit y mediante electroforesis en gel de agarosa. La preparación de bibliotecas y la secuenciación de genes 16S e ITS se realizaron en una plataforma Illumina MiSeq, generando un mínimo de 25,000 lecturas de 300 pares de bases por tipo de amplicón para cada muestra. Los bloqueadores pPNA y mPNA se usaron para evitar la amplificación y secuenciación del ADN de las plantas.

2.2 Procesamiento de secuencias de amplicones de las regiones 16S e ITS

El acceso al protocolo detallado y los métodos bioinformáticos utilizados para procesar y analizar los genes 16S y las secuencias de ITS está disponible en GitHub (https://github.com/ajaraservin/mezcal). Los datos de amplicones del microbioma del pulque se obtuvieron de lecturas crudas disponibles públicamente (Rocha-Arriaga et al. 2020) para ser procesados usando el mismo protocolo de análisis computacional. La evaluación de calidad se realizó en todas las bibliotecas 16S utilizando FastQC v0.12.0 (https://www.bioinformatics.babraham.ac.uk/projects/fastqc/), y se descartaron 8 muestras debido a la baja cantidad de lecturas obtenidas. Esto también llevó a que una de las destilerías fuera excluida del análisis, ya que no quedaron muestras de esta. El ensamblador CASPER v0.8.2 se utilizó para unir las lecturas de extremos pareados de la región V4 (Kwon et al. 2014). Para identificar secuencias quiméricas, se utilizó el script identify_chimeric_seqs.py de QIIME con el fragmento BLAST de ChimeraSlayer (Caporaso et al. 2010). Todas las muestras se concatenaron y se agruparon en Unidades Taxonómicas Operativas (OTUs) usando un umbral de identidad del 97% con cd-hit-est (Li y Godzik 2006). La asignación taxonómica se realizó utilizando los scripts de QIIME (Caporaso et al. 2010) contra la base de datos Silva v138 (Yilmaz et al. 2014). Tras el cribado de quimeras, secuencias únicas y contaminantes, las secuencias restantes se alinearon y se construyó un árbol filogenético usando FastTreeMP v2.1.11 (Price et al. 2009).

Las lecturas de extremos pareados de ITS se unieron utilizando el ensamblador CASPER y luego se sometieron a un control de calidad mediante fastq_quality_filter (Q<20) del FASTX-Toolkit (http://hannonlab.cshl.edu/fastx_toolkit/). Se descartaron 12 bibliotecas de ITS debido al bajo

número de lecturas, lo que resultó en que dos destilerías no se incluyeran en el análisis. Después del ensamblaje con PANDASEQ v2.11 y la agrupación con una identidad del 97% con cd-hit-est, la base de datos UNITE v8.3 (Nilsson et al. 2019) se utilizó para la asignación taxonómica hasta el nivel de especie. Todas las secuencias no fúngicas y quiméricas se eliminaron usando los scripts parallel_identify_chimeric_seqs.py y filter_otus_from_otu_table.py de QIIME (Caporaso et al. 2010).

2.3 Análisis de diversidad y estadísticos

Las OTUs se usaron para analizar tanto la diversidad alfa como la diversidad beta de las muestras de destilados de agave y las de pulque. Se utilizaron los paquetes de R Phyloseq (McMurdie y Holmes 2013), ggplot2 (Wickham 2016), vegan (Oksanen et al. 2022), y los paquetes predeterminados de R v4.2.2 (Team 2021) para el análisis. Se calcularon los índices de diversidad Observada, Chao1, Shannon y Simpson para evaluar la diversidad alfa, mientras que los perfiles de abundancia taxonómica se obtuvieron agrupando a diferentes niveles taxonómicos. Los dendrogramas se construyeron usando el método hclust. Se empleó el Análisis de Coordenadas Principales restringido (CAP) en una matriz UniFrac no ponderada (Lozupone y Knight 2005) de secuencias del gen 16S rRNA para evaluar la diversidad beta de acuerdo con las distintas variables. Del mismo modo, se utilizó una matriz de similitud de Jaccard (Knight et al. 2018) de secuencias ITS para el método de ordenación PcoA. La UniFrac no se utilizó para el análisis de ITS debido a la dificultad de alinear las secuencias ITS (Halwachs et al. 2017). Ambas matrices fueron evaluadas utilizando la función estadística ANalysis Of SIMilarity (ANOSIM) (Varsos et al. 2016). Las siguientes variables fueron evaluadas: Destilería (Distillery_ID), Clima (ClimateGroup_GK), Temperatura del tanque (Tank_temperatureC), Etapa de fermentación

(Fermentation_range), Especie de agave (Agave_speciesname), Región (Region), Material del tanque (Tank_material). Los nombres de las variables en paréntesis son los nombres empleados en la Tabla Suplementaria 1, donde se proporciona una descripción detallada de cada variable. La abundancia diferencial de OTUs se estimó utilizando el paquete DESeq2 de R (Love et al. 2014), como se hace de manera rutinaria (Halwachs et al. 2017). Finalmente, se utilizó Geographic Distance Matrix Generator v1.2.3 (Ersts) para generar una matriz de distancia geográfica para todas las muestras de fermentos de agave. Esta matriz de distancia geográfica se utilizó para realizar una prueba de Mantel (Xia y Sun 2017), utilizando una matriz de distancia UniFrac no ponderada para bacterias y una matriz de similitud de Jaccard para hongos. Los métodos bioinformáticos y estadísticos detallados están disponibles en GitHub (/github.com/ajaraservin/mezcal).

3 RESULTADOS

3.1 Una colección de muestras de mosto de agave de diversas regiones biogeográficas del país

Este trabajo forma parte de la investigación realizada por el consorcio YeastGenomesMx, un grupo de laboratorios enfocados en la diversidad genómica de levaduras y otros microorganismos en México. En esta caracterización del microbioma de las fermentaciones de agave, nos enfocamos en 99 tanques de fermentación de 42 destilerías, de las cuales 67 utilizaron *Agave angustifolia*, la especie más comúnmente empleada para las bebidas tradicionales de agave (Tabla Suplementaria 1). La especie *A. tequilana*, utilizada para producir tequila, es la más cultivada; sin embargo, debido a que la producción de tequila es principalmente industrial y depende de inóculos de levaduras comerciales, no fue considerada en este trabajo. Al enfocar el análisis en destilerías que

usan *A. angustifolia*, pudimos evaluar el impacto de otras variables, como la ubicación de la destilería, en el microbioma de las fermentaciones. Dado que existen regiones donde no se emplea *A. angustifolia*, también incluimos fermentaciones de otras ocho especies de agave y mezclas de dos o tres especies para la producción de destilados combinados. Las especies utilizadas en estas destilerías incluyen *Agave americana*, *A. durangensis*, *A. inaequidens*, *A. karwinski*, *A. mapisaga*, *A. potatorum*, *A. rhodacantha* y *A. salmiana* (Tabla Suplementaria 1). Esta variedad de fermentaciones nos permitió probar el posible efecto del sustrato vegetal en la diversidad microbiana.

Las 42 destilerías se encuentran en 31 municipios de los estados de Durango, Guanajuato, Michoacán, Oaxaca, Puebla, Tamaulipas, Jalisco y Sonora. En los seis primeros estados, la bebida producida es conocida como mezcal, mientras que en Jalisco se llama raicilla y en Sonora, bacanora. Las tres bebidas cuentan con su propia denominación de origen, aunque el proceso general de producción es similar. Además, varios productores usan el término "destilado de agave" para no adherirse a las normas oficiales mexicanas (NOM) de cada denominación de origen. Las dos destilerías más distantes muestreadas están separadas por más de 2,000 km en línea recta, desde 29°52' Norte y 109°33' Oeste hasta 16° Norte y 96°31' Oeste. La altitud de las destilerías varió considerablemente, desde menos de 500 metros sobre el nivel del mar hasta más de 2,500 metros, y abarcaron climas que van desde tropicales hasta semiáridos. La Figura 1 muestra la ubicación geográfica de las destilerías analizadas en este estudio y algunas de sus características geográficas.

Todas, excepto tres de las destilerías incluidas en este estudio, usaron hornos de piedra o ladrillo enterrados, y los corazones de agave cocidos se trituraron de manera manual, con molinos accionados por animales o empleando molinos motorizados. No se midieron ni la temperatura ni la duración de la cocción en cada sitio, pero se han reportado temperaturas promedio de 120 °C y duraciones de tres días a más de una semana (Durán-García et al. 2007). Los tanques muestreados abarcaron tiempos de fermentación desde el momento en que se formuló la fermentación hasta su madurez completa, cuando el mosto se transfería al alambique para la destilación. Dado que la duración total de la fermentación varía entre destilerías, esto significó que muestreamos tanques que llevaban menos de un día de fermentación hasta otros que habían fermentado durante casi dos meses. El material de los tanques fue diverso, incluyendo madera, plástico, arcilla, acero, cemento e incluso cuero vacuno, aunque la madera fue el material más común, seguido del plástico, entre las destilerías muestreadas. Las características específicas de las destilerías y los tanques de fermentación de las muestras secuenciadas se detallan en la Tabla Suplementaria 1.

3.2 Diversidad bacteriana en fermentaciones de agave en México

En este estudio, evaluamos y comparamos la comunidad bacteriana de las fermentaciones de agave en todo México mediante la secuenciación de metabarcodes 16S. De las 99 muestras colectadas, 91 se secuenciaron exitosamente, generando un total de 10,438,060 lecturas 16S pareadas. Las lecturas fueron filtradas por calidad y ensambladas, obteniendo 4,887,502 secuencias con un promedio de 53,708.81 ± 15,371.02 secuencias por muestra (Tabla Suplementaria 2). Estas secuencias fueron luego agrupadas utilizando un umbral de identidad del 97% en 10,093 Unidades Taxonómicas Operativas (OTUs), que correspondieron a 888 géneros dentro de un total de 29 filos procariontes distintos. En promedio, cada muestra contenía una riqueza de 1,002.08 ± 316.47

OTUs. La riqueza promedio esperada de Chao1 fue de 1,690.17 ± 500.67 OTUs, lo que mostró que nuestro esfuerzo cubrió gran parte de los OTUs bacterianos esperados. Los índices de diversidad Shannon-Weiner (H') y Simpson (D) promedio en las muestras fueron de 3.26 ± 0.66 y 0.86 ± 0.12, respectivamente.

A nivel de filo, la composición de las comunidades procariotas fue similar en todas las muestras y estuvo compuesta predominantemente por Firmicutes (71.82%), Proteobacteria (14%), Actinobacteriota (4.82%), Bacteroidota (4.02%), Verrucomicrobiota (0.76%) y Patescibacteria (0.65%). De las 71 clases bacterianas identificadas, Bacilli dominó (6,802 OTUs; 68.33%), seguido por Alphaproteobacteria (971 OTUs; 9.75%), Gammaproteobacteria (971 OTUs; 9.75%), Actinobacteria (412 OTUs; 4.13%), Bacteroidia (399 OTUs; 4.00%), Clostridia (373 OTUs; 3.74%) y Parcubacteria (46 OTUs; 0.46%). Los diez géneros más abundantes fueron *Weissella* (948 OTUs; 7,125.88 ± 59,886 lecturas por muestra), *Paucilactobacillus* (258 OTUs; 7,083.38 ± 43,347.79 lecturas por muestra), *Lentilactobacillus* (749 OTUs; 2,598.77 ± 22,071.50 lecturas), *Leuconostoc* (678 OTUs; 6,051.23 ± 59,767.16 lecturas por muestra), *Oenococcus* (266 OTUs; 4,222.21 ± 28,871.62 lecturas), *Lactiplantibacillus* (509 OTUs; 4,372.17 ± 21,691.45 lecturas por muestra), *Liquorilactobacillus* (373 OTUs; 10,639.12 ± 67,213.38 lecturas por muestra), *Lacticaseibacillus* (587 OTUs; 116.16 ± 384.15 lecturas por muestra), *Acetobacter* (236 OTUs; 1,351.17 ± 4,614.47 lecturas por muestra) y *Secundilactobacillus* (572 OTUs; 5,501.06 ± 35,204.32 lecturas). Todos estos géneros, excepto *Acetobacter*, son bacterias ácido-lácticas. También observamos una considerable diversidad dentro de los géneros identificados. Por ejemplo, el 50.22% de los géneros (446) estaban representados por más de una OTU y el 10.13% (90 géneros) por diez o más (Tabla Suplementaria 2).

Los géneros más abundantes descritos anteriormente, excepto *Acetobacter*, estuvieron presentes en todas las destilerías muestreadas. En general, hubo 15 géneros presentes en todos los sitios analizados, todas ellas bacterias ácido-lácticas, que podrían considerarse el microbioma bacteriano central de las fermentaciones de agave (Tabla 1). Si se usa una definición más flexible de comunidad núcleo, además de estos 15, hubo otros 11 géneros presentes en el 80% o más de las destilerías y en las siete regiones productoras de bebidas de agave (Tabla 1). La mayoría (73%) de los 26 géneros eran bacterias ácido-lácticas. El perfil de abundancia taxonómica de procariotas se muestra en la Figura 2 y la lista completa de géneros se encuentra en la Tabla Suplementaria 2.

De los 734 géneros bacterianos que se pudieron clasificar, el 96% no habían sido asociados previamente con fermentaciones tradicionales de agave utilizando métodos microbiológicos de cultivo, incluida una investigación reciente que empleó metabarcoding en una sola destilería (Colón-González et al. 2024, Kirchmayr et al. 2024). Del microbioma bacteriano central, 12 (46%) géneros no habían sido reportados en estudios previos (Tabla 1). Es importante señalar que algunos de estos géneros se definieron recientemente, lo que podría explicar por qué no se habían reportado previamente en fermentaciones de agave. Además, de los 31 géneros que habían sido reportados en fermentaciones previas, nuestro estudio solo omitió a *Zymomonas*. Sin embargo, *Zymomonas* no está presente como un género en la base de datos de referencia de 16S más actual, en la cual las secuencias que pertenecían a este género han sido reasignadas a cinco géneros de la familia Sphingomonadaceae, cuatro de los cuales identificamos en las fermentaciones de agave. En conjunto, nuestros resultados muestran que el esfuerzo de muestreo contribuyó considerablemente

a la comprensión de la composición procariota en las fermentaciones tradicionales de agave, una parte de este microbioma que había sido mayormente ignorada.

Tabla 1. Microbioma núcleo bacteriano y fúngico de las fermentaciones tradicionales de agave

Género bacteriano[1]	OTUs[2]	Destilerías[3]	Especie fúngica[1]	OTUs[2]	Destilerías[3]
Weissella	948	100	*Saccharomyces cerevisiae*	109	100
Paucilactobacillus#	258	100	*Pichia spp.*	24	93
Lentilactobacillus#	749	100	*Torulaspora delbrueckii*	24	100
Leuconostoc	678	100	*Kluyveromyces marxianus*	14	95
Oenococcus	266	100	*Penicillium polonicum*#	16	83
Lactiplantibacillus	509	100	*Pichia mandshurica*	26	88
Liquorilactobacillus#	373	100	*Hanseniaspora spp.*	15	90
Lacticaseibacillus	587	100	*Pichia kluyveri*	346	93
Acetobacter	236	98	*Zygosaccharomyces bisporus*	13	88
Secundilactobacillus	572	100	*Mycosphaerella tassiana*#	11	83
Gluconobacter	210	98	*unidentified unidentified*	32	95
Levilactobacillus	349	100	*Zygosaccharomyces bailii*	6	88
Komagataeibacter	163	95	*Aureobasidium pullulans*#	8	80
*ANPR**#	34	88			
Latilactobacillus#	129	100			
Schleiferilactobacillus#	125	100			
Limosilactobacillus#	169	98			

Lactobacillus	122	98
Companilactobacillus[#]	144	100
Pediococcus	194	100
Bacillus	56	95
Loigolactobacillus[#]	44	100
Geobacillus	21	85
Bifidobacterium[#]	27	85
Ligilactobacillus[#]	65	98
Enterococcus[#]	42	85

[1]Los taxones fueron incluidos si estaban presentes en el 80% o más de las destilerías y en las siete regiones productoras. Se muestran de arriba hacia abajo en orden descendente de abundancia relativa, como en la Figura 2. [2]Número de OTUs clasificadas en el taxón. [3]Porcentaje de destilerías en las que se identificó el taxón.
[*] *ANPR, Allorhizobium-Neorhizobium-Pararhizobium-Rhizobium*
[#] Géneros/especies que no se habían asociado previamente con fermentaciones de agave.

3.3 Diversidad fúngica en fermentaciones de agave en México

Para caracterizar el microbioma fúngico de las fermentaciones de agave, realizamos la secuenciación de amplicones de la región ITS a partir de las muestras de mosto. Un total de 87 muestras fueron secuenciadas exitosamente, generando un total de 10,199,202 lecturas ITS pareadas. Las lecturas se ensamblaron y filtraron por calidad, obteniendo 2,135,405 secuencias ensambladas con un promedio de 24,544.8 ± 11,380.3 lecturas por muestra (Tabla Suplementaria 2). Agrupando las secuencias a una distancia filogenética del 3%, identificamos un total de 1,118 OTUs de ITS. En promedio, se observaron 92.85 ± 39.72 OTUs en las 87 muestras, mientras que la riqueza promedio esperada (Chao1) fue de 153.92 ± 70.44. Al igual que con los procariotas, nuestro esfuerzo cubrió la mayoría de la diversidad esperada por muestra. La diversidad en cada muestra fue considerablemente menor que para las bacterias, con un índice de diversidad de Shannon-Weiner (H’) promedio de 1.20 ± 0.74 y un índice de Simpson (D) promedio de 0.44 ±

0.27. La gran mayoría de los OTUs fúngicos pertenecieron al filo Ascomycota (1,035 OTUs; 92.57%), aunque también identificamos Basidiomycota (40 OTUs; 3.57%), Mucoromycota (7 OTUs; 0.62%), Chytridiomycota (2 OTUs; 0.17%), Mortierellomycota (1 OTU; 0.08%) y Rozellomycota (1 OTU; 0.08%). Once OTUs fúngicos (0.98%) permanecieron sin identificar incluso a nivel de filo.

Los 1,118 OTUs ITS identificados se distribuyeron en 325 especies fúngicas. Las diez especies de hongos más abundantes fueron *Saccharomyces cerevisiae* (109 OTUs; 16,793.54 ± 11,282.19 lecturas por muestra), *Pichia mandshurica* (26 OTUs; 1,159.20 ± 4,523.03 lecturas por muestra), una especie no identificada de *Pichia* (24 OTUs; 1,155.11 ± 3,443.42 lecturas por muestra), *Torulaspora delbrueckii* (24 OTUs; 896.75 ± 1,997.14 lecturas por muestra), *Penicillium polonicum* (16 OTUs; 686.39 ± 2,436.22 lecturas por muestra), *Kluyveromyces marxianus* (14 OTUs; 624.43 ± 1,062.53 lecturas por muestra), *Zygosaccharomyces bisporus* (13 OTUs; 535.22 ± 1,069.18 lecturas por muestra), una especie no identificada de *Hanseniaspora* (15 OTUs; 514.40 ± 953.92 lecturas por muestra), *Pichia kluyveri* (346 OTUs; 496.59 ± 441.87 lecturas por muestra), y otra especie no identificada (32 OTUs; 295.34 ± 1,648.88 lecturas por muestra). Estas especies representaron el 94.26% del número total de lecturas. Como en el caso de muchos géneros procariotas, se observó una gran diversidad intraespecífica; 81 (23.5%) especies contaban con más de dos OTUs y 19 (5.5%) con más de diez (Tabla Suplementaria 2).

Solo *S. cerevisiae* y *T. delbrueckii* estuvieron presentes en todas las destilerías, pero hubo otras once especies fúngicas identificadas en el 80% o más de los sitios y en las siete regiones productoras. Estas 13 especies podrían considerarse como el microbioma fúngico central de las

fermentaciones de agave (Tabla 1). Una de las especies del núcleo no pudo ser identificada ni siquiera a nivel de filo, pero el resto son todos ascomicetos. También se detectaron numerosas especies raras, con 125 encontradas en un solo tanque de fermentación, y se identificaron 35 y 34 OTUs fúngicos que no pudieron asignarse a ningún género o especie, respectivamente. El perfil de abundancia taxonómica de hongos se muestra en la Figura 2 y la lista completa de especies se encuentra en la Tabla Suplementaria 2.

En total, 234 (91.0%) de las especies fúngicas identificadas no se habían asociado anteriormente con fermentaciones tradicionales de agave (Colón-González et al. 2024). Del núcleo de especies, *P. polonicum*, *M. tassiana* y *A. pullulans* no habían sido reportadas previamente. Por otro lado, 23 especies previamente aisladas mediante métodos basados en cultivo no fueron identificadas en nuestro estudio, aunque once de ellas no están incluidas en la base de datos ITS utilizada aquí. A nivel de género, el 90.7% de los 184 géneros identificados en este estudio no habían sido asociados con fermentaciones tradicionales de agave y solo omitimos tres géneros previamente aislados (15%), todos Basidiomicetos, y uno de ellos no está incluido en la base de datos ITS que empleamos. En conjunto, estos resultados muestran que nuestro esfuerzo enriquece la comprensión de las comunidades fúngicas de las fermentaciones tradicionales de agave, añadiendo un considerable número de especies y géneros que no se habían asociado con este medio ambiente.

3.4 La diversidad microbiana solo se explica a nivel de destilería

Para identificar las variables geográficas, climáticas y de producción que influyen en la composición microbiana de los tanques de fermentación, analizamos la diversidad beta generando dendrogramas basados en las similitudes de los microbiomas de las muestras (Figura 3). Las

variables más importantes asociadas con cada muestra pueden observarse en la Figura 3 como barras de color junto a los dendrogramas. La única agrupación en la que tanto la ordenación fúngica como la bacteriana fue estadísticamente significativa (ANOSIM) fue la correspondiente a la destilería (r = 0.5967, P = 0.0001 para bacterias; r = 0.1703, P = 0.0315 para hongos). Esto también se aprecia en los dendrogramas, donde las muestras pertenecientes a la misma destilería suelen aparecer en los mismos grupos (Figura 3). Ninguna otra variable fue estadísticamente significativa para hongos, mientras que la etapa de fermentación, el clima y la región productora también fueron significativas para las bacterias (r = 0.2567, P = 0.006; r = 0.2768, P = 0.0001 y r = 0.1949, P = 0.0111, respectivamente).

Dada la agrupación observada por destilería, realizamos una prueba de Mantel para evaluar si existe una correlación entre la distancia geográfica y la diversidad microbiana de las fermentaciones. El valor r resultante para las bacterias fue de 0.2321 con una significancia de 0.001, mientras que para los hongos fue de 0.08247 y 0.101, respectivamente. Para las bacterias, estos resultados mostraron que la composición microbiana de dos destilerías cercanas es más similar que la de destilerías más distantes, lo cual concuerda con los resultados de ANOSIM al evaluar las muestras por destilería y región. En contraste, para los hongos, el efecto que observamos por destilería (ANOSIM) no se observó en todas las escalas espaciales, sino solo a nivel de destilerías. En resumen, observamos que la destilería es un importante determinante de las comunidades tanto bacterianas como fúngicas de los tanques de fermentación, y, en el caso de las bacterias, la fase de fermentación, el clima y la región productora también fueron factores determinantes generales.

3.5 Dinámicas diferentes de las comunidades bacteriana y fúngica durante la fermentación de *Agave*

Nuestros análisis generales de todas las destilerías revelaron una asociación significativa entre la etapa de fermentación y la composición bacteriana de las fermentaciones de agave (r = 0.2567, P = 0.006, ANOSIM). Sin embargo, esto no se observó para los hongos, lo cual fue algo sorprendente dado que en otras fermentaciones abiertas empleadas para la producción de otras bebidas se han observado sucesiones ecológicas (Pinto et al. 2015, Boynton y Greig 2016, Liu et al. 2020, Martiniuk et al. 2023). Es importante señalar que el análisis de ANOSIM previo se realizó con los tiempos de fermentación de todos los tanques muestreados. Para investigar esto más a fondo, nos enfocamos en dos destilerías específicas para las que teníamos muestras en diferentes tiempos de fermentación (Figura 4). Las muestras de estas dos destilerías se agruparon en tres etapas relativas de fermentación, cada una representando un tercil del tiempo total de fermentación (Inicial, Media y Final), y los cambios en la diversidad se evaluaron mediante medidas de diversidad alfa. Las tendencias observadas hacia una disminución en la diversidad de OTUs bacterianos y fúngicos al final de la fermentación no fueron estadísticamente significativas (Figura 4 y Figura Suplementaria 1). Sin embargo, en el caso de las bacterias, la estructura de la comunidad fue influenciada significativamente por la etapa de fermentación, como lo reflejan los resultados de ANOSIM al considerar estas dos destilerías (r = 0.4608, P = 0.0128). En contraste, ANOSIM mostró que las comunidades fúngicas son más uniformes a lo largo del proceso de fermentación (r = 0.02222, P = 0.4398), como había sido sugerido por el análisis de todas las muestras de fermentación.

Para determinar si existían taxones bacterianos y fúngicos específicos enriquecidos en las diferentes etapas de fermentación, realizamos un análisis con DESeq2. Aunque este método fue

desarrollado para evaluar la expresión diferencial de genes, puede utilizarse de manera general para estimar diferencias en datos de recuento obtenidos mediante secuenciación de alto rendimiento, como se ha hecho anteriormente en análisis de metabarcoding (Halwachs et al. 2017). Para las bacterias, solo encontramos géneros enriquecidos al comparar las etapas inicial o media con la etapa final, y el número de géneros enriquecidos en las etapas inicial o media fue más del doble que en la etapa final (Figura Suplementaria 2). *Allorhizobium*, *Komagataeibacter*, *Lentilactobacillus*, *Leuconostoc*, *Levilactobacillus*, *Liquorilactobacillus*, *Oenococcus* y *Paucilactobacillus* se enriquecieron tanto en las fases inicial como media de la fermentación. Los dos primeros géneros pertenecen a las Proteobacteria y el resto a Firmicutes. *Companilactobacillus*, *Lactobacillus* y *Limosilactobacillus*, todos Firmicutes, se enriquecieron en la etapa final en comparación con las otras dos etapas. Todos los géneros enriquecidos diferencialmente forman parte del componente bacteriano central de las fermentaciones de agave.

En el caso de los hongos, solo hubo especies enriquecidas al comparar las etapas inicial y final de fermentación con la fase media (Figura Suplementaria 2). *K. marxianus* se enriqueció al inicio de la fermentación en comparación con la fase media, mientras que *Dekkera anomala* mostró el enriquecimiento inverso. *Citeromyces matritensis*, *Hanseniaspora osmophila* y *T. delbrueckii* se enriquecieron en la fase media de la fermentación en comparación con la etapa final, mientras que *Aspergillus niger* y *Neurospora terricola* mostraron el patrón inverso. Todas estas especies son ascomicetas, y tanto *K. marxianus* como *T. delbrueckii* forman parte del componente fúngico central de las fermentaciones de agave.

La asociación estadísticamente significativa entre la composición bacteriana y la etapa de fermentación sugiere la posible ocurrencia de una sucesión ecológica a lo largo de la fermentación de agave, en concordancia con los hallazgos de otros procesos de fermentación (Pinto et al. 2015, Boynton y Greig 2016, Liu et al. 2020, Martiniuk et al. 2023). Sin embargo, en las fermentaciones de agave, los cambios en la composición fúngica pueden ser más sutiles en comparación con otros sistemas. Por lo tanto, serán necesarios análisis adicionales con un tamaño de muestra mayor y específicamente diseñados para comparar diferentes etapas, con el fin de detectar tales variaciones sutiles en la composición fúngica de estas fermentaciones.

3.6 Géneros específicos bacterianos se enriquecen en fermentaciones de ciertos agaves dentro de una destilería

Aunque la agrupación por especie de agave utilizada en la fermentación no fue estadísticamente significativa al considerar todas las muestras, incluimos una destilería donde se llevaban a cabo fermentaciones de diferentes especies de agave en paralelo (D13). Esto ofreció una oportunidad única para evaluar la influencia de la planta utilizada como sustrato, ya que todas las demás variables eran similares entre los tanques. Como puede observarse en los dendrogramas de la Figura 3, los microbiomas bacterianos de las muestras de *Agave potatorum* de esta destilería se agruparon entre sí, mientras que los de otros agaves, incluyendo varias muestras de *A. angustifolia*, no lo hicieron. No observamos una agrupación clara por especie de agave para los microbiomas fúngicos en esta destilería. Mediante un análisis con DESeq2, identificamos 16 OTUs bacterianos que se enriquecieron diferencialmente entre los microbiomas de las muestras de *A. potatorum* y el resto de los agaves (Figura Suplementaria 3). Todos, excepto uno, pertenecen a Firmicutes, y varios forman parte del microbioma bacteriano central. Es importante señalar que, al realizar el

mismo análisis pero comparando las fermentaciones de *A. angustifolia* con el resto de las muestras, solo un OTU se enriqueció diferencialmente. Aunque se necesitan más experimentos para comprender mejor la influencia de la especie de agave en el microbioma, nuestros hallazgos sugieren que la composición bioquímica específica de las plantas podría tener efectos determinantes sobre la composición microbiana de las fermentaciones de agave, al menos en el caso de *A. potatorum*.

3.7 El microbioma de las fermentaciones de destilados de agave y de pulque son considerablemente diferentes

El pulque es una bebida tradicional mexicana producida a partir de la fermentación del aguamiel de agave. A diferencia de lo que se hace para producir destilados de agave, el aguamiel no se cuece para la producción de pulque, y la bebida es una fermentación directa sin destilación. El microbioma involucrado en las fermentaciones de pulque ha sido bien caracterizado utilizando enfoques metagenómicos (Rocha-Arriaga et al. 2020, Astudillo-Melgar et al. 2023) y, dado que el sustrato de fermentación es similar, aguamiel frente a mosto de agave cocido, es un excelente punto de comparación para comprender mejor las comunidades microbianas involucradas en la producción de bebidas destiladas de agave. Para el análisis de pulque, utilizamos 1,399,910 lecturas pareadas de 16S e ITS previamente secuenciadas (Rocha-Arriaga et al. 2020). Estas secuencias se generaron a partir de seis muestras de pulque con un promedio de 74,882.16 ± 336.61 lecturas por muestra para bacterias y 10,782.16 ± 280.71 lecturas para hongos. En promedio, las comunidades bacterianas de las muestras de pulque tuvieron un mayor número de OTUs observadas (3,105.5 ± 277.09, Tabla Suplementaria 3) que las muestras de bebidas espirituosas de agave (1,002.08 ± 316.47). En concordancia, las comunidades bacterianas de las fermentaciones

para destilados de agave son menos diversas que las de pulque, mostrando un índice de Shannon (H’) de 3.26 ± 0.66, mientras que la comunidad de pulque mostró 3.64 ± 0.51. La dominancia de especies, evaluada a través del índice de Simpson, presentó valores similares en las fermentaciones de destilados de agave (0.86 ± 0.12) y en las muestras de pulque (D = 0.85 ± 0.06). De manera similar, los microbiomas de pulque mostraron en promedio una mayor cantidad de OTUs observadas de ITS (114.33 ± 98.82) en comparación con las muestras de bebidas destiladas de agave (92.85 ± 39.5). Para los hongos, tanto los índices de Shannon como de Simpson también fueron más elevados para las comunidades de las muestras de pulque (H’ = 2.18 ± 0.21; D = 0.80 ± 0.03 en pulque, frente a H’ = 1.20 ± 0.74; D = 0.44 ± 0.27 en destilados de agave).

En total, se compartieron 100 géneros bacterianos y 49 especies fúngicas entre las muestras de pulque y de destilados de agave. A pesar de estas similitudes, los microbiomas de los dos tipos de fermentaciones son claramente diferentes. Las fermentaciones de destilados de agave tienen 788 géneros bacterianos y 282 especies fúngicas exclusivas, mientras que las fermentaciones de pulque solo tienen 9 y 12, respectivamente (Tablas Suplementarias 2 y 3). Además, los análisis de diversidad beta tanto bacteriana como fúngica mediante ordenación multivariante utilizando CAP mostraron una separación evidente entre los microbiomas de pulque y de destilados de agave (Figura 5). En general, cada ordenación bacteriana y fúngica explica el 19% y el 40% de la varianza observada para bacterias y hongos, respectivamente, y muestra que las comunidades de las fermentaciones de bebidas espirituosas de agave difieren más que las muestras de pulque (Figura 5). Además de los taxones que solo se encontraron en un tipo de fermentación, el análisis DESeq2 identificó un total de 31 géneros bacterianos presentes en ambos ambientes, pero sobrerrepresentados en las fermentaciones de destilados de agave, y solo ocho sobrerrepresentados

en el pulque. De manera similar, doce especies de hongos se enriquecieron significativamente en las fermentaciones de destilados de agave, mientras que ocho especies se sobrerrepresentaron en el pulque. En resumen, nuestra comparación mostró diferencias importantes entre los microbiomas de las fermentaciones para destilados de agave y el pulque, a pesar de que ambos utilizan agaves como materia prima.

4 DISCUSIÓN

4.1 Un análisis a nivel nacional de la diversidad microbiana en fermentaciones tradicionales de agave

La producción de destilados de agave es de importancia cultural y comercial central para México. En las últimas décadas, los aromas y sabores de estas bebidas han ganado reconocimiento mundial, hasta el punto de que otros países, como Sudáfrica, Australia y Kenia, también han comenzado a producir destilados de agave (Smith 2017, Yan et al. 2020). La mayor parte del enfoque en esta actividad ha sido en la variedad de planta y las prácticas de destilación empleadas, dejando de lado a los microorganismos que realizan la fermentación (Arellano-Plaza et al. 2022). Por ejemplo, las normas oficiales mexicanas de la denominación de origen del mezcal (NOM-070-SCFI-2016) especifican la ubicación geográfica, el estado de madurez y el modo de transporte de las plantas de agave, pero en cuanto a la fermentación solo menciona una amplia variedad de materiales posibles para las tinas. Los microorganismos que llevan a cabo la fermentación son en gran medida desconocidos para las destilerías tradicionales, en las cuales no se emplean inóculos y el proceso, presumiblemente, es realizado por bacterias y levaduras autóctonas del entorno circundante. Para comprender mejor estas comunidades microbianas, en este estudio caracterizamos la composición

fúngica y procariota de una colección de mostos de fermentación a nivel nacional empleando un enfoque de metabarcoding. A pesar de la amplia gama de características geoclimáticas y prácticas de producción de las destilerías, observamos un conjunto central de géneros bacterianos y especies fúngicas prevalentes en la mayoría de las fermentaciones (Tabla 1). Las bacterias ácido-lácticas fueron las más comunes, aunque también identificamos un número considerable de bacterias acéticas. En el caso de los hongos, los ascomicetos fueron, con diferencia, los más ubicuos y abundantes, dado que otros filos representaron menos del 10% del total de OTUs. Estos hallazgos concuerdan con los microorganismos que se han aislado en estudios previos de este tipo de fermentaciones, que en conjunto abarcaron cerca de quince destilerías (Colón-González et al. 2024). Es importante señalar que nuestro estudio reveló una gran diversidad intragenérica entre procariotas y variabilidad intraespecífica entre hongos, con los géneros y especies más abundantes abarcando múltiples OTUs.

Nuestro trabajo amplió considerablemente la lista de microorganismos asociados con fermentaciones tradicionales de agave. Esto es especialmente cierto para los procariotas, que apenas habían sido considerados antes: cerca del 98% de los géneros bacterianos que identificamos no habían sido descritos en este entorno. En el caso de los hongos, más del 90% de los géneros y especies detectados tampoco habían sido aislados de fermentaciones de agave. Varios de los taxones que consideramos aquí como el núcleo de los microbiomas (Tabla 1) no se habían aislado en estudios previos, lo que muestra la importancia de nuestro esfuerzo para comprender mejor estas comunidades. Para los procariotas, esto era esperable, ya que solo unos pocos estudios previos se habían centrado en ellos (Escalante-Minakata et al. 2008, Narvaez-Zapata et al. 2010, Kirchmayr et al. 2017). En el caso de los hongos, fue sorprendente encontrar a *P. polonicum*, *M.*

tassiana y *A. pullulans* como taxones prevalentes en fermentaciones de agave. Se sabe que todas estas especies están ampliamente distribuidas en diversos entornos, y *P. polonicum* y *A. pullulans* se han utilizado para aplicaciones biotecnológicas (Chi et al. 2009, Ding et al. 2013). Por otro lado, *P. polonicum* suele asociarse con el deterioro de alimentos y *M. tassiana* pertenece a un gran género de hongos fitopatógenos (Barr 1958, Duduk et al. 2014). Será interesante definir si estas tres especies contribuyen de manera positiva o negativa a la dinámica de fermentación y a las propiedades organolépticas en la producción de destilados de agave.

Observamos la ausencia de géneros bacterianos y fúngicos que habían sido identificados en estudios previos, lo cual concuerda con nuestras estimaciones de diversidad que sugieren que aún queda por determinar una fracción de las comunidades microbianas en cada destilería. Es probable que los microorganismos identificados previamente y no encontrados en el presente estudio sean especies más raras con distribuciones focalizadas, ya que no se habían reportado en más de un estudio. Los cambios en la taxonomía de los microorganismos también explicaron algunas de las discrepancias con informes previos, como fue el caso de *Zymomonas*. Al comparar la diversidad microbiana de las fermentaciones de agave con la de otros alimentos y bebidas fermentadas, la diversidad bacteriana (índice de Shannon) es cercana a la diversidad promedio en otras fermentaciones, mientras que la diversidad fúngica es más parecida a los entornos menos diversos, como las fermentaciones de vino (Leech et al. 2020, Rocha-Arriaga et al. 2020, Kharnaior y Tamang 2023, Martiniuk et al. 2023, Qi et al. 2023). En general, nuestro trabajo establece las bases para una comprensión más completa de la composición y diversidad de las comunidades microbianas responsables de la fermentación necesaria para producir destilados tradicionales de agave.

4.2 La composición de las comunidades microbianas en las fermentaciones de agave se define a nivel de destilería

Al igual que en otros sistemas de fermentación abierta, se espera que los factores climáticos y las prácticas de elaboración determinen la composición microbiana de las fermentaciones de agave en diferentes escalas geográficas. Por ejemplo, se esperaría que las fermentaciones dentro de una misma región productora tengan microbiomas más similares entre sí que con los de una región diferente. Nuestro trabajo ofreció la primera oportunidad para investigar tales efectos en todo el país, ya que los esfuerzos previos se habían centrado principalmente en una o dos destilerías y empleaban métodos de aislamiento que dificultaban la comparación directa entre estudios. Las diferencias en los microbiomas fueron observadas para bacterias y hongos solo al comparar a nivel de destilería. Esto sugiere que el microbioma de las fermentaciones de agave se define localmente, en primer lugar, por las características y prácticas específicas de cada sitio de producción. Las pocas diferencias que encontramos en los microbiomas de tanques de etapas de fermentación distintas y de diferentes especies de agave dentro de una sola destilería están en concordancia con esta sugerencia; las condiciones dentro de la destilería son más determinantes que otros factores.

Nuestros resultados también mostraron que los componentes fúngico y bacteriano de las fermentaciones de agave están influenciados de manera diferente por factores ambientales. Aparte de las observaciones a nivel de destilería, se observaron diferencias significativas en los microbiomas bacterianos de muestras de diferentes etapas de fermentación, climas y regiones productoras, pero no en el componente fúngico de las fermentaciones de agave. En general, observamos menos diversidad en el microbioma fúngico, con 13 especies que forman un componente fúngico central presente en casi todas las fermentaciones muestreadas en todo el país

(Tabla 1), y estas especies mostraron una considerable diversidad intraespecífica. Resta por ver si la diversidad fúngica dentro de las especies se correlaciona mejor con la distribución geográfica. Nuestros hallazgos contrastan en cierta medida con lo que se ha observado en los microbiomas de las fermentaciones de vino, específicamente en el microbioma fúngico. Se ha informado que las comunidades bacterianas y fúngicas de bodegas en California y Portugal son específicas de las denominaciones de origen, especialmente en las primeras etapas de fermentación (Pinto et al. 2015, Bokulich et al. 2016). Es importante señalar que las regiones vinícolas analizadas son considerablemente más pequeñas que el área donde se ubican las fermentaciones de agave muestreadas en este estudio y que los microorganismos endógenos de la planta no se eliminan mediante una etapa de cocción. También se han encontrado diferencias entre los microbiomas de viñedos individuales, de manera similar a lo que observamos entre destilerías de agave, pero además hubo diferencias entre las regiones vitivinícolas. Se ha sugerido que estas diferencias contribuyen a las características específicas de los vinos de las denominaciones de origen mencionadas. Las diferencias en la composición química entre los mostos de agave y uva también pueden contribuir a las variaciones en los patrones microbianos entre los dos entornos de fermentación. Por ejemplo, el rango de contenido de etanol es más estrecho en las fermentaciones de agave, ya que la concentración final solo alcanza aproximadamente el 6% (Colón-González et al. 2024), mientras que en el vino suele superar el 10%.

Es posible que los microbiomas de las fermentaciones utilizadas en la producción de destilados de agave se asemejen más a los involucrados en la fermentación de miso en Japón. Un estudio reciente reveló que la latitud tiene un impacto relativamente pequeño en las comunidades fúngicas y bacterianas de estas fermentaciones, mientras que el efecto de la planta de fermentación era fuerte

incluso cuando los hongos eran inoculados por los productores (Koide et al. 2024). Los esfuerzos para estandarizar la producción de miso empleando inóculos fúngicos específicos pasan por alto la resiliencia de los microbiomas específicos de cada planta fermentadora. De manera similar, los intentos de estandarizar las fermentaciones de agave pueden enfrentar obstáculos debido a las diversas comunidades microbianas moldeadas por las condiciones y prácticas locales de cada destilería.

La amplia gama de características geográficas y climáticas que abarcan los sitios de producción de destilados de agave, sumada a la gran variedad de diferencias culturales en las prácticas de producción, hacen de las fermentaciones de agave un entorno muy diverso. Por lo tanto, es posible que se necesite analizar un mayor número de destilerías y tanques de fermentación para identificar mejor los factores que definen las comunidades microbianas de las fermentaciones de agave. Sin embargo, nuestro trabajo representa el esfuerzo más exhaustivo hasta la fecha para comprender estos microbiomas y proporciona información valiosa sobre los taxones específicos que los constituyen. Además, la colección de mostos de agave que generamos ofrece la oportunidad de realizar futuras caracterizaciones químicas detalladas de las fermentaciones, lo cual podría revelar otros factores que se asocian con el microbioma.

4.3 El tiempo de fermentación, la especie de agave y las prácticas de producción influyen en los microbiomas de las fermentaciones de agave

Observamos una tendencia hacia una disminución en la diversidad alfa tanto de OTUs bacterianos como fúngicos al final de la fermentación, aunque las diferencias no fueron estadísticamente significativas (Figura Suplementaria 1). La prueba ANOSIM reveló una influencia significativa

de la etapa de fermentación en la estructura de las comunidades procariotas en las fermentaciones de agave, destacando una clara diferenciación en la diversidad beta bacteriana a lo largo de las etapas. Además, ciertos grupos bacterianos se enriquecieron en distintas etapas de fermentación. La disminución observada en la diversidad hacia el final de la fermentación y la correlación significativa entre la diversidad bacteriana y la etapa de fermentación coinciden con hallazgos de otros procesos de fermentación (Pinto et al. 2015, Bokulich et al. 2016). En contraste, las comunidades fúngicas parecieron ser más uniformes a lo largo de las fermentaciones. En general, nuestros resultados están en concordancia con el estudio de diversidad de levaduras en fermentaciones tradicionales de agave que realizamos recientemente empleando métodos basados en cultivo (Gallegos-Casillas et al. 2023). Este trabajo, centrado en las comunidades de levaduras, reveló cambios en la composición a lo largo del tiempo, aunque la diversidad alfa no mostró diferencias estadísticas entre las etapas. Sin embargo, a diferencia de los resultados de metabarcoding presentados aquí, los análisis previos de diversidad beta de levaduras sí mostraron una disminución estadísticamente significativa en función de la fase de fermentación, aunque la magnitud del cambio fue pequeña (Gallegos-Casillas et al. 2023).

La disminución observada en la diversidad hacia el final de la fermentación y la correlación estadísticamente significativa entre la diversidad bacteriana y la etapa de fermentación coinciden con hallazgos de otros procesos de fermentación alcohólica (Costa et al. 2015, Pinto et al. 2015, Huang et al. 2023). Sin embargo, en las fermentaciones de agave, los cambios en la composición fúngica pueden ser más sutiles en comparación con otras fermentaciones (Gallegos-Casillas et al. 2023). Para lograr una comprensión más completa de la sucesión ecológica en las fermentaciones espontáneas de agave, se necesitan análisis adicionales con un mayor tamaño de muestra y

específicamente diseñados para comparar diferentes etapas. Estas investigaciones adicionales descubrirán detalles más precisos sobre la dinámica microbiana y arrojarán luz sobre los complejos procesos ecológicos que ocurren durante la fermentación de agave.

Se considera que la especie de planta utilizada para producir destilados de agave es uno de los factores más influyentes que contribuyen al sabor y aroma de estas bebidas. Existen destilados elaborados a partir de variedades de agave, como *A. potatorum*, que son muy valorados por los consumidores y cuyos precios son considerablemente más altos. Es posible que algunos de los atributos aportados por la variedad específica de agave provengan indirectamente de la influencia que los componentes de la planta tienen en la comunidad microbiana responsable de la fermentación. Al analizar fermentaciones de diferentes plantas de agave dentro de la misma destilería, observamos ciertos géneros bacterianos asociados con fermentaciones de *A. potatorum*. Claramente, se necesita más trabajo, incluyendo estrategias de muestreo específicamente diseñadas, para comprender mejor esta asociación, pero nuestros hallazgos sugieren la posibilidad de que los efectos de la especie de planta en las propiedades organolépticas de los destilados puedan ocurrir al determinar la composición de los microbiomas de las fermentaciones.

Las prácticas de producción son otro factor determinante de la calidad de los destilados de agave. Por ejemplo, la destilación en ollas de barro se utiliza en algunas productoras porque se piensa que añade sabores específicos, a pesar de ser un sistema de destilación menos eficiente. En cuanto a una práctica que podría afectar el proceso de fermentación, no observamos diferencias estadísticamente significativas en las comunidades microbianas cuando las muestras se agruparon según el material de la tina de fermentación y el método de molienda utilizado. En cuanto a otras

variables que no se encontraron asociadas con la composición microbiana, es posible que se necesite un conjunto de muestras más amplio para detectar tales efectos, dada la complejidad y diversidad del sistema. Observamos una diferencia importante entre las bacterias y los hongos de las fermentaciones de pulque y destilados de agave. A nivel fundamental, la materia prima utilizada para producir ambas bebidas son los oligosacáridos de las plantas de agave. Sin embargo, en la producción de destilados de agave, los compuestos de la planta han sido sometidos a altas temperaturas durante períodos considerables. Además de romper los oligosacáridos y transformar químicamente otros componentes de la planta, este paso presumiblemente elimina todos los microorganismos de la planta y su entorno, y la inoculación del mosto de agave por microorganismos presumiblemente comienza después de la cocción, cuando el vapor se enfría. Un estudio previo sugirió que las herramientas en la destilería podrían servir como reservorios de levaduras para cada nueva fermentación (Lachance 1995), pero se necesita más investigación para comprender la dinámica ecológica de estos microorganismos. Nuestros hallazgos sugieren que la cocción de los corazones de agave tiene importantes implicaciones para la composición de las comunidades microbianas de las fermentaciones de agave.

4.4 Conclusiones finales

Hasta donde sabemos, el trabajo presentado aquí representa el análisis más completo de las comunidades bacterianas y fúngicas involucradas en las fermentaciones abiertas necesarias para producir destilados tradicionales de agave. Identificamos cientos de especies que no habían sido asociadas previamente con este entorno y detectamos diferencias locales en los microbiomas de las destilerías muestreadas. Las diferencias locales sugieren que la suma de factores históricos y ambientales, así como las prácticas de producción de cada destilería, determinan la composición

microbiana de las fermentaciones de agave. A su vez, el microbioma específico de cada destilería puede contribuir a las propiedades del destilado de agave correspondiente, añadiendo al terroir de estas bebidas. Dado el ritmo acelerado con el que está cambiando la producción de destilados de agave debido a la creciente demanda, las comunidades microbianas identificadas aquí servirán como base para comprender mejor y preservar las fermentaciones en este proceso tradicional único.

LEYENDAS DE LAS FIGURAS

Figura 1. Distribución y características de las destilerías tradicionales de agave. Para poder comparar los datos con nuestro trabajo anterior, el formato del mapa y los diagramas de caja de las características climáticas son los mismos que en (Gallegos-Casillas et al. 2023). Sin embargo, los datos en las figuras corresponden a las propiedades de las destilerías utilizadas en el trabajo actual. A) Mapa de México que muestra la ubicación geográfica de las 42 destilerías (puntos negros) de las cuales se tomaron muestras de 99 tanques de fermentación en la región productora correspondiente, que se indican como polígonos en diferentes colores de fondo. Las regiones productoras se definieron según Aguirre (2006), principalmente en función de las especies de agave y las prácticas de producción empleadas. Las regiones Oeste I y Oeste II se consideraron una sola región (Oeste) en la publicación original, pero después de consultar con los autores, la región se dividió para tener en cuenta las diferencias observadas en las prácticas de producción de las destilerías de esas áreas (Gallegos-Casillas et al. 2023). B) Características climáticas de las ubicaciones de los tanques de fermentación de agave. Arriba a la izquierda, temperatura media anual, arriba a la derecha, isotermalidad, abajo a la derecha, precipitación media anual, y abajo a la izquierda, altura sobre el nivel medio del mar.

Figura 2. Composición bacteriana y fúngica de las comunidades microbianas en las fermentaciones de agave. Mapas de calor que muestran la abundancia relativa de los géneros bacterianos (panel izquierdo) y las especies fúngicas (panel derecho) identificados en cada destilería. Solo se muestran los 100 taxones más abundantes, ordenados de arriba a abajo según su abundancia relativa total. La abundancia relativa se refiere al número de lecturas de un género/especie en relación con el número total de lecturas en la destilería o en todas las destilerías. Los taxones considerados como parte del microbioma central (presente en el 80% o más de las destilerías y en todas las siete regiones productoras, Tabla 1) se marcan con un asterisco rojo. Los números a la derecha de cada mapa de calor muestran el número total de destilerías en las que se identificó el taxón. Todos los taxones bacterianos y fúngicos identificados se reportan en la Tabla Suplementaria 2.

Figura 3. Análisis de la diversidad microbiana de las fermentaciones de agave y su relación con las prácticas de producción y características geográficas. Los dendrogramas muestran la similitud de los microbiomas bacterianos (panel izquierdo) y fúngicos (panel derecho) de las fermentaciones muestreadas. Se utiliza el mismo color de fuente para muestras de la misma destilería. Se empleó la distancia UniFrac para bacterias y la similitud de Jaccard para hongos. Las características de producción y geoclimáticas más importantes asociadas con cada muestra se muestran en barras verticales de colores a la derecha de cada dendrograma. De izquierda a derecha, se muestra el índice de diversidad de Shannon (H'), la especie de agave utilizada (Agave), la etapa de fermentación expresada como porcentaje del tiempo total de fermentación (Time), el material del tanque (Tank), la región productora como se describe en la Figura 1 (Region) y el grupo climático (Climate). La composición de las fermentaciones con mezclas de plantas de agave (ensambles) se describe en la Tabla Suplementaria 1. Se utilizaron los grupos climáticos de Köppen modificados por E. García (Amaro 2004). Los cuatro grupos climáticos que

abarcan las destilerías son tropical [Aw], subtropical [(A)C], semiárido [BS] y templado [C(w)]. Si una variable afecta la composición microbiana, las muestras en el cladograma deberían agruparse según esa variable. Solo observamos agrupación clara por destilería. Los resultados de ANOSIM mostraron que solo la destilería se asocia con la composición fúngica, mientras que la destilería, la etapa de fermentación, el clima y la región se asociaron con la composición bacteriana.

Figura 4. Cambios en la comunidad microbiana a lo largo del tiempo durante la fermentación tradicional de agave. Abundancia relativa de los géneros bacterianos (panel izquierdo) y las especies fúngicas (panel derecho) identificados en cada una de las tres fases de fermentación en dos destilerías que tenían fermentaciones en tiempos diferentes cuando se tomaron las muestras. Solo se muestran los 100 taxones más abundantes, ordenados de arriba a abajo según su abundancia relativa total. La abundancia relativa se refiere al número de lecturas de un género/especie en relación con el número total de lecturas en la destilería o en todas las destilerías. Las flechas rojas indican taxones que fueron identificados como enriquecidos diferencialmente entre dos etapas de fermentación según el análisis DESeq2, como se detalla en la Figura Suplementaria 2.

Figure 5. Los microbiomas del pulque y de las fermentaciones de destilados de agave son considerablemente diferentes. Análisis de diversidad beta de comunidades bacterianas (panel izquierdo) y fúngicas (panel derecho) en pulque (puntos rojos) y fermentaciones utilizadas para la producción de destilados de agave (puntos azules). Para las bacterias se utilizó PCA con una matriz de distancia UniFrac no ponderada y para los hongos PcoA basada en una matriz de similitud de Jaccard.

Figura Suplementaria 1. Análisis de diversidad de las diferentes etapas de fermentación en dos destilerías. Estimadores de diversidad de las tres etapas de fermentación analizadas en dos destilerías específicas que tenían tanques de diferentes tiempos cuando se realizó el muestreo.

Figura Suplementaria 2. Análisis DESeq2 de las diferentes etapas de fermentación en dos destilerías. Resultados de DESeq2 de la comparación de las tres etapas de fermentación diferentes a nivel de OTU de las mismas dos destilerías. Solo se muestran las comparaciones en las que hubo OTUs enriquecidos diferencialmente.

Figura Suplementaria 3. Análisis DESeq2 en diferentes especies de agave dentro de una misma destilería.

Tabla Suplementaria 1. Características de los tanques de fermentación donde se recolectaron las muestras.

Tabla Suplementaria 2. Taxones bacterianos y fúngicos identificados en fermentaciones tradicionales de agave. Esta tabla contiene cuatro pestañas, dos para procariotas (16S) y dos para hongos (ITS). Para cada tipo de microorganismo hay una lista de todos los OTUs identificados (sufijo "_OTU") y de todos los géneros/especies identificados (sufijos "_Genus" y "_Species"). En las pestañas de OTU, para cada muestra, las primeras cuatro filas proporcionan el número total de lecturas en bruto (“Raw_paired_sequences”), el número total de lecturas ensambladas (“Assembled_sequences”), el número total de lecturas ensambladas y filtradas (“Filtered_sequences”) y el número total de OTUs (“Total_OTUs”).

Tabla Suplementaria 3. OTUs y taxones bacterianos y fúngicos identificados en pulque.

Material Suplementario 1. Traducción al español del artículo.

DISPONIBILIDAD DE DATOS

Todos los datos han sido depositados en NCBI bajo el BioProject PRJNA1085712.

AGRADECIMIENTOS

Estamos profundamente agradecidos con todos los productores de destilados de agave en los estados de Durango, Guanajuato, Michoacán, Oaxaca, Puebla, Tamaulipas, Jalisco y Sonora, quienes gentilmente participaron en este trabajo al proporcionar acceso a muestras y compartir su conocimiento. Los detalles sobre los agradecimientos relacionados con la ayuda en el trabajo de campo y el muestreo por parte del consorcio YeastGenomesMx pueden encontrarse en (Gallegos-Casillas et al. 2023). Agradecemos a Susana Ruiz-Castro, Porfirio Gallegos-Casillas, J. Abraham Avelar-Rivas y Luis F. García-Ortega por la asistencia técnica.

FINANCIAMIENTO

Este trabajo fue financiado por el Consejo Nacional de Humanidades, Ciencias y Tecnologías de México (Conahcyt, con los apoyos FORDECYT-PRONACES/103000/2020 y CF-2023-G-695) y el Programa de Apoyo a Proyectos de Investigación e Innovación Tecnológica DGAPA-UNAM (apoyo IN230420). AJ-S fue estudiante de doctorado en el Posgrado en Ciencias Biológicas - UNAM con una beca de Conahcyt (765278), SIJ-C fue estudiante de maestría del Posgrado en Biotecnología de Plantas en Cinvestav con una beca de Conahcyt (755846), y EM recibió una beca del Conahcyt para realizar una estancia sabática (I0200/111/2024); los patrocinadores no tuvieron ningún rol en el diseño del estudio, la recolección y análisis de datos, la decisión de publicación ni en la preparación del manuscrito.

CONTRIBUCIONES DE LOS AUTORES

Conceptualización: LM, AD, AH-L, EM. Metodología: AJ-S, LDA, SIJ-S. Trabajo de campo: LM, AD, AH-L, EM. Investigación, trabajo de laboratorio: SIJ-S, AE-J, IB. Investigación, análisis formal: AJ-S, LDA, EM. Visualización: AJ-S, LDA, EM. Adquisición de financiamiento: LM, AD, AH-L, EM. Supervisión: AH-L, EM. Redacción, borrador original: AJ-S, EM. Redacción, revisión y edición: LDA, LM, AD, AH-L. Todos los autores leyeron y aprobaron la versión final del manuscrito.

CONFLICTO DE INTERESES

Los autores declaran que la investigación se llevó a cabo en ausencia de relaciones comerciales o financieras que pudieran interpretarse como un posible conflicto de intereses.

VERSIONES DEL MANUSCRITO

La primera versión de este manuscrito fue publicada como preprint en bioRxiv (Jara-Servin A. et al., 2024).

REFERENCIAS

Aguirre, X., Illsley, C., Larson, J. 2006. "Dulce semblanza de los mezcales del Altiplano y del Balsas." México Desconocido **352**: 36–45.

Amaro, E. G. 2004. Modificaciones al Sistema de Clasificación Climática de Köppen. Mexico, Instituto de Geografía-UNAM.

Arellano-Plaza, M., J. B. Paez-Lerma, N. O. Soto-Cruz, M. R. Kirchmayr and A. G. Mathis. 2022. "Mezcal Production in Mexico: Between Tradition and Commercial Exploitation." Frontiers in Sustainable Food Systems **6**.

Astudillo-Melgar, F., G. Hernández-Chávez, M. E. Rodríguez-Alegría, F. Bolívar and A. Escalante (2023) "Analysis of the Microbial Diversity and Population Dynamics during the Pulque Fermentation Process." Fermentation **9** DOI: 10.3390/fermentation9040342.

Barr, M. E. 1958. "Life History Studies of Mycosphaerella-Tassiana and M-Typhae." Mycologia **50**(4): 501-513.

Bokulich, N. A., T. S. Collins, C. Masarweh, G. Allen, H. Heymann, S. E. Ebeler and D. A. Mills. 2016. "Associations among Wine Grape Microbiome, Metabolome, and Fermentation Behavior Suggest Microbial Contribution to Regional Wine Characteristics." Mbio **7**(3).

Boynton, P. J. and D. Greig. 2016. "Species richness influences wine ecosystem function through a dominant species." Fungal Ecology **22**: 61-72.

Cabrera-Toledo, D., E. Mendoza-Galindo, N. Larranaga, A. Herrera-Estrella, M. Vásquez-Cruz and T. Hernández-Hernández (2022) "Genomic and Morphological Differentiation of Spirit Producing Agave angustifolia Traditional Landraces Cultivated in Jalisco, Mexico." Plants **11** DOI: 10.3390/plants11172274.

Caporaso, J. G., J. Kuczynski, J. Stombaugh, K. Bittinger, F. D. Bushman, E. K. Costello, N. Fierer, A. G. Pena, J. K. Goodrich, J. I. Gordon, G. A. Huttley, S. T. Kelley, D. Knights, J. E. Koenig, R. E. Ley, C. A. Lozupone, D. McDonald, B. D. Muegge, M. Pirrung, J. Reeder, J. R. Sevinsky, P. J. Turnbaugh, W. A. Walters, J. Widmann, T. Yatsunenko, J. Zaneveld and R. Knight. 2010. "QIIME allows analysis of high-throughput community sequencing data." Nat Methods **7**(5): 335-336.

Chi, Z., F. Wang, Z. Chi, L. Yue, G. Liu and T. Zhang. 2009. "Bioproducts from Aureobasidium pullulans, a biotechnologically important yeast." Appl Microbiol Biotechnol **82**(5): 793-804.

Colón-González, M., X. Aguirre-Dugua, M. G. Guerrero-Osornio, J. A. Avelar-Rivas, A. DeLuna, E. Mancera and L. Morales. 2024. "Thriving in Adversity: Yeasts in the Agave Fermentation Environment."

Colunga-GarciaMarin, P., I. Torres-García, A. Casas, C. Figueredo-Urbina, S. Rangel-Landa, A. Lemus, O. Vargas-Ponce, D. Cabrera-Toledo, D. Zizumbo-Villarreal, X. Aguirre-Dugua, L. Eguiarte and G. Galván (2017). Los agaves y las prácticas mesoamericanas de aprovechamiento, manejo y domesticación 1**:** 273-308.

Costa, O. Y., B. M. Souto, D. D. Tupinamba, J. C. Bergmann, C. M. Kyaw, R. H. Kruger, C. C. Barreto and B. F. Quirino. 2015. "Microbial diversity in sugarcane ethanol production in a Brazilian distillery using a culture-independent method." J Ind Microbiol Biotechnol **42**(1): 73-84.

Ding, G. Z., J. Liu, J. M. Wang, L. Fang and S. S. Yu. 2013. "Secondary metabolites from the endophytic fungi Penicillium polonicum and Aspergillus fumigatus." J Asian Nat Prod Res **15**(5): 446-452.

Duduk, N., M. Vasic and I. Vico. 2014. "First Report of Penicillium polonicum Causing Blue Mold on Stored Onion (Allium cepa) in Serbia." Plant Dis **98**(10): 1440.

Durán-García, H. M., E. J. González-Galván and P. Matadamas-Ortiz. 2007. "Mechanization process in the production of mezcal." Journal of Food Agriculture & Environment **5**(3-4): 32-35.

Eberhard, D. M., G. F. Simons and C. D. Fennig. 2020. Ethnologue: Languages of the World. Dallas, SIL International.

Ersts, P. J. I. "Geographic Distance Matrix Generator(version 1.2.3)." Retrieved 2023-2-16 from http://biodiversityinformatics.amnh.org/open_source/gdmg.

Escalante-Minakata, P., H. P. Blaschek, A. P. Barba de la Rosa, L. Santos and A. De Leon-Rodriguez. 2008. "Identification of yeast and bacteria involved in the mezcal fermentation of Agave salmiana." Lett Appl Microbiol **46**(6): 626-630.

Farjon, A. 1996. "Biodiversity of Pinus (Pinaceae) in Mexico: Speciation and palaeo-endemism." Botanical Journal of the Linnean Society **121**(4): 365-384.

Gallegos-Casillas, P., L. F. Garcia-Ortega, A. Espinosa-Cantu, J. A. Avelar-Rivas, C. G. Torres-Lagunes, A. Cano-Ricardez, A. M. Garcia-Acero, S. Ruiz-Castro, M. Flores-Barraza, A. Castillo, F. Gonzalez-Zozaya, A. Delgado-Lemus, F. Molina-Freaner, C. Jacques-Hernandez, A. Hernandez-Lopez, L. Delaye, X. Aguirre-Dugua, M. R. Kirchmayr, L. Morales, E. Mancera and A. DeLuna. 2023. "Yeast diversity in open agave fermentations across Mexico." Yeast.

Halwachs, B., N. Madhusudhan, R. Krause, R. H. Nilsson, C. Moissl-Eichinger, C. Hogenauer, G. G. Thallinger and G. Gorkiewicz. 2017. "Critical Issues in Mycobiota Analysis." Front Microbiol **8**: 180.

Huang, P., Y. Jin, M. Liu, L. Peng, G. Yang, Z. Luo, D. Jiang, J. Zhao, R. Zhou and C. Wu. 2023. "Exploring the Successions in Microbial Community and Flavor of Daqu during Fermentation Produced by Different Pressing Patterns." Foods **12**(13).

Kharnaior, P. and J. P. Tamang. 2023. "Microbiome and metabolome in home-made fermented soybean foods of India revealed by metagenome-assembled genomes and metabolomics." Int J Food Microbiol **407**: 110417.

Kirchmayr, M. R., L. E. Segura-García, P. Lappe-Oliveras, R. Moreno-Terrazas, M. de la Rosa and A. G. Mathis. 2017. "Impact of environmental conditions and process modifications on microbial diversity, fermentation efficiency and chemical profile during the fermentation of in Oaxaca." Lwt-Food Science and Technology **79**: 160-169.

Kirchmayr, M. R., L. Vital-López, M. Arellano-Plaza, A. C. Gschaedler, C. P. Larralde-Corona and J. A. Narváez-Zapata. 2024. "Bacterial community inferred by metagenomic analysis in an artisanal mezcal distillery in Guerrero, Mexico." Biocatalysis and Agricultural Biotechnology **58**: 103159.

Knight, R., A. Vrbanac, B. C. Taylor, A. Aksenov, C. Callewaert, J. Debelius, A. Gonzalez, T. Kosciolek, L. I. McCall, D. McDonald, A. V. Melnik, J. T. Morton, J. Navas, R. A. Quinn, J. G. Sanders, A. D. Swafford, L. R. Thompson, A. Tripathi, Z. Z. Xu, J. R. Zaneveld, Q. Zhu, J. G. Caporaso and P. C. Dorrestein. 2018. "Best practices for analysing microbiomes." Nat Rev Microbiol **16**(7): 410-422.

Koide, R. T., M. Kanauchi and Y. Hashimoto. 2024. "Variation Among Japanese Miso Breweries in Indoor Microbiomes is Mainly Ascribed to Variation in Type of Indoor Surface." Current Microbiology **81**(2).

Kwon, S., B. Lee and S. Yoon. 2014. "CASPER: context-aware scheme for paired-end reads from high-throughput amplicon sequencing." BMC Bioinformatics **15**(9): S10.

Lachance, M. A. 1995. "Yeast communities in a natural tequila fermentation." Antonie Van Leeuwenhoek **68**(2): 151-160.

Leech, J., R. Cabrera-Rubio, A. M. Walsh, G. Macori, C. J. Walsh, W. Barton, L. Finnegan, F. Crispie, O. O'Sullivan, M. J. Claesson and P. D. Cotter. 2020. "Fermented-Food Metagenomics Reveals Substrate-Associated Differences in Taxonomy and Health-Associated and Antibiotic Resistance Determinants." mSystems **5**(6).

Li, W. and A. Godzik. 2006. "Cd-hit: a fast program for clustering and comparing large sets of protein or nucleotide sequences." Bioinformatics **22**(13): 1658-1659.

Liu, D., Q. L. Chen, P. Z. Zhang, D. L. Chen and K. S. Howell. 2020. "The Fungal Microbiome Is an Important Component of Vineyard Ecosystems and Correlates with Regional Distinctiveness of Wine." Msphere **5**(4).

Love, M. I., W. Huber and S. Anders. 2014. "Moderated estimation of fold change and dispersion for RNA-seq data with DESeq2." Genome Biol **15**(12): 550.

Lozupone, C. and R. Knight. 2005. "UniFrac: a new phylogenetic method for comparing microbial communities." Appl Environ Microbiol **71**(12): 8228-8235.

Machuca Chávez, C. P. 2018. El vino de cocos en la Nueva España : historia de una transculturación en el siglo XVII. Zamora, Michoacán, El Colegio de Michoacán.

Martiniuk, J. T., J. Hamilton, T. Dodsworth and V. Measday. 2023. "Grape-associated fungal community patterns persist from berry to wine on a fine geographical scale." Fems Yeast Research **23**.

McMurdie, P. J. and S. Holmes. 2013. "phyloseq: an R package for reproducible interactive analysis and graphics of microbiome census data." PLoS One **8**(4): e61217.

Narvaez-Zapata, J. A., R. A. Rojas-Herrera, I. C. Rodriguez-Luna and C. P. Larralde-Corona. 2010. "Culture-independent analysis of lactic acid bacteria diversity associated with mezcal fermentation." Curr Microbiol **61**(5): 444-450.

Nilsson, R. H., K. H. Larsson, A. F. S. Taylor, J. Bengtsson-Palme, T. S. Jeppesen, D. Schigel, P. Kennedy, K. Picard, F. O. Glockner, L. Tedersoo, I. Saar, U. Koljalg and K. Abarenkov. 2019. "The UNITE database for molecular identification of fungi: handling dark taxa and parallel taxonomic classifications." Nucleic Acids Res **47**(D1): D259-D264.

Nixon, K. C. (2006). Global and Neotropical Distribution and Diversity of Oak (genus Quercus) and Oak Forests. Ecology and Conservation of Neotropical Montane Oak Forests. M. Kappelle. Berlin, Heidelberg, Springer Berlin Heidelberg**:** 3-13.

Nolasco-Cancino, H., J. A. Santiago-Urbina, C. Wacher and F. Ruíz-Terán. 2018. "Predominant Yeasts During Artisanal Mezcal Fermentation and Their Capacity to Ferment Maguey Juice." Frontiers in Microbiology **9**.

Ojeda-Linares, C., G. D. Alvarez-Rios, C. J. Figueredo-Urbina, L. A. Islas, P. Lappe-Oliveras, G. P. Nabhan, I. Torres-Garcia, M. Vallejo and A. Casas. 2021. "Traditional Fermented Beverages of Mexico: A Biocultural Unseen Foodscape." Foods **10**(10).

Oksanen, J., G. L. Simpson, F. G. Blanchet, R. Kindt, P. Legendre, P. R. Minchin, R. B. O'Hara, P. Solymos, M. H. H. Stevens, E. Szoecs, H. Wagner, M. Barbour, M. Bedward, B. Bolker, D. Borcard, G. Carvalho, M. Chirico, M. De Caceres, S. Durand, H. B. A. Evangelista, R. FitzJohn, M. Friendly, B. Furneaux, G. Hannigan, M. O. Hill, L. Lahti, D. McGlinn, M.-H. Ouellette, E. Ribeiro Cunha, T. Smith, A. Stier, C. J. F. Ter Braak and J. Weedon. 2022. "vegan: Community Ecology Package."

Pinto, C., D. Pinho, R. Cardoso, V. Custódio, J. Fernandes, S. Sousa, M. Pinheiro, C. Egas and A. C. Gomes. 2015. "Wine fermentation microbiome: a landscape from different Portuguese wine appellations." Frontiers in Microbiology **6**.

Price, M. N., P. S. Dehal and A. P. Arkin. 2009. "FastTree: computing large minimum evolution trees with profiles instead of a distance matrix." Mol Biol Evol **26**(7): 1641-1650.

Qi, Y., G. O. Bruni and K. T. Klasson. 2023. "Microbiome Analysis of Sugarcane Juices and Biofilms from Louisiana Raw Sugar Factories." Microbiol Spectr **11**(3): e0434522.

Rocha-Arriaga, C., A. Espinal-Centeno, S. Martinez-Sanchez, J. Caballero-Perez, L. D. Alcaraz and A. Cruz-Ramirez. 2020. "Deep microbial community profiling along the fermentation process of pulque, a biocultural resource of Mexico." Microbiol Res **241**: 126593.

Serra Puche, M. C. and J. s. C. Lazcano Arce. 2016. El mezcal, una bebida prehispánica : estudios etnoarqueológicos / Mari Carmen Serra Puche, Jesús Carlos Lazcano Arce. México, Distrito Federal, Universidad Nacional Autónoma de México, Instituto de Investigaciones Antropológicas.

Smith, G. F. 2017. "Producing organic alcohol and a tequila-like liquor from
L. (Asparagaceae subfam. Agavoideae / Agavaceae) at Graaff-Reinet in the Eastern Cape Province of South Africa: challenges to establish an industry based on a naturalised, alien succulent." Bradleya **35**: 15-32.

Sohail, M., M. J. Palma-Martinez, A. Y. Chong, C. D. Quinto-Cortes, C. Barberena-Jonas, S. G. Medina-Munoz, A. Ragsdale, G. Delgado-Sanchez, L. P. Cruz-Hervert, L. Ferreyra-Reyes, E. Ferreira-Guerrero, N. Mongua-Rodriguez, S. Canizales-Quintero, A. Jimenez-Kaufmann, H. Moreno-Macias, C. A. Aguilar-Salinas, K. Auckland, A. Cortes, V. Acuna-Alonzo, C. R. Gignoux, G. L. Wojcik, A. G. Ioannidis, S. L. Fernandez-Valverde, A. V. S. Hill, M. T. Tusie-Luna, A. J. Mentzer, J. Novembre, L. Garcia-Garcia and A. Moreno-Estrada. 2023. "Mexican Biobank advances population and medical genomics of diverse ancestries." Nature **622**(7984): 775-783.

Suazo-Ortuño, I., A. Ramírez-Bautista and J. Alvarado-Díaz (2023). Amphibians and Reptiles of Mexico: Diversity and Conservation. Mexican Fauna in the Anthropocene. R. W. Jones, C. P. Ornelas-García, R. Pineda-López and F. Álvarez. Cham, Springer International Publishing**:** 105-127.

Team, R. C. 2021. "R: A Language and Environment for Statistical Computing."

Trejo, L., V. Limones, G. Peña, E. Scheinvar, O. Vargas-Ponce, D. Zizumbo-Villarreal and P. Colunga-GarcíaMarín. 2018. "Genetic variation and relationships among agaves related to the production of Tequila and Mezcal in Jalisco." Industrial Crops and Products **125**: 140-149.

Varsos, C., T. Patkos, A. Oulas, C. Pavloudi, A. Gougousis, U. Z. Ijaz, I. Filiopoulou, N. Pattakos, E. Vanden Berghe, A. Fernandez-Guerra, S. Faulwetter, E. Chatzinikolaou, E. Pafilis, C. Bekiari, M. Doerr and C. Arvanitidis. 2016. "Optimized R functions for analysis of ecological community data using the R virtual laboratory (RvLab)." Biodivers Data J(4): e8357.

Wickham, H. (2016). ggplot2 : Elegant Graphics for Data Analysis. Use R!,. Cham, Springer International Publishing : Imprint: Springer,**:** 1 online resource (XVI, 260 pages 232 illustrations, 140 illustrations in color.

Xia, Y. and J. Sun. 2017. "Hypothesis Testing and Statistical Analysis of Microbiome." Genes Dis **4**(3): 138-148.

Yan, X. Y., K. R. Corbin, R. A. Burton and D. K. Y. Tan. 2020. "Agave: A promising feedstock for biofuels in the water-energy-food environment (WEFE) nexus." Journal of Cleaner Production **261**.

Yilmaz, P., L. W. Parfrey, P. Yarza, J. Gerken, E. Pruesse, C. Quast, T. Schweer, J. Peplies, W. Ludwig and F. O. Glockner. 2014. "The SILVA and "All-species Living Tree Project (LTP)" taxonomic frameworks." Nucleic Acids Res **42**(Database issue): D643-648.
